# A fully automated home cage for long-term continuous phenotyping of mouse cognition and behavior

**DOI:** 10.1016/j.crmeth.2023.100532

**Published:** 2023-07-13

**Authors:** Hinze Ho, Nejc Kejzar, Hiroki Sasaguri, Takashi Saito, Takaomi C. Saido, Bart De Strooper, Marius Bauza, Julija Krupic

**Affiliations:** 1 Department of Physiology, Development and Neuroscience, University of Cambridge, Cambridge, UK; 2 Laboratory for Proteolytic Neuroscience, RIKEN Brain Science Institute, Wako, Japan; 3 Department of Neurology and Neurological Science, Graduate School of Medicine, Tokyo Medical and Dental University, Tokyo, Japan; 4 Department of Neurocognitive Science, Institute of Brain Science, Nagoya City University Graduate School of Medical Sciences, Nagoya, Japan; 5 UK-Dementia Research Institute, University College London, London, UK; 6 Department of Neurosciences, Leuven Brain Institute, KU Leuven, Leuven, Belgium; 7 VIB Center for Brain & Disease Research, Leuven, Belgium; 8 Sainsbury Wellcome Centre, University College London, London, UK

**Keywords:** mouse behavioural cognitive testing, home-cage system, machine learning, automated classification, longitudinal monitoring, Alzheimer's disease

## Abstract

Automated home-cage monitoring systems present a valuable tool for comprehensive phenotyping of natural behaviors. However, current systems often involve complex training routines, water or food restriction, and probe a limited range of behaviors. Here, we present a fully automated home-cage monitoring system for cognitive and behavioral phenotyping in mice. The system incorporates T-maze alternation, novel object recognition, and object-in-place recognition tests combined with monitoring of locomotion, drinking, and quiescence patterns, all carried out over long periods. Mice learn the tasks rapidly without any need for water or food restrictions. Behavioral characterization employs a deep convolutional neural network image analysis. We show that combined statistical properties of multiple behaviors can be used to discriminate between mice with hippocampal, medial entorhinal, and sham lesions and predict the genotype of an Alzheimer’s disease mouse model with high accuracy. This technology may enable large-scale behavioral screening for genes and neural circuits underlying spatial memory and other cognitive processes.

## Highlights

• Fully automated execution of T-maze and novel object recognition test in home cages
• Rapid task learning with no food or water restrictions
• Testing has been done over long periods
• Performance accuracy comparable to analogous standard tests

## Motivation

Automated phenotyping of mouse behavior is essential for improving standardization and increasing throughput, especially in the context of translational research, which may involve large numbers of different mouse groups. Unsupervised automated phenotyping of mouse cognitive functions, such as learning and memory, has been particularly challenging. Moreover, ideally the testing should be animal-friendly and ethologically relevant. Here, we present the home monitoring system, which includes fully automated implementation of T-maze, novel object recognition, and object-in-place tests, as well as monitoring of locomotion activities. All testing is carried out with no food or water restrictions.

## Graphical abstract

**Figure:**
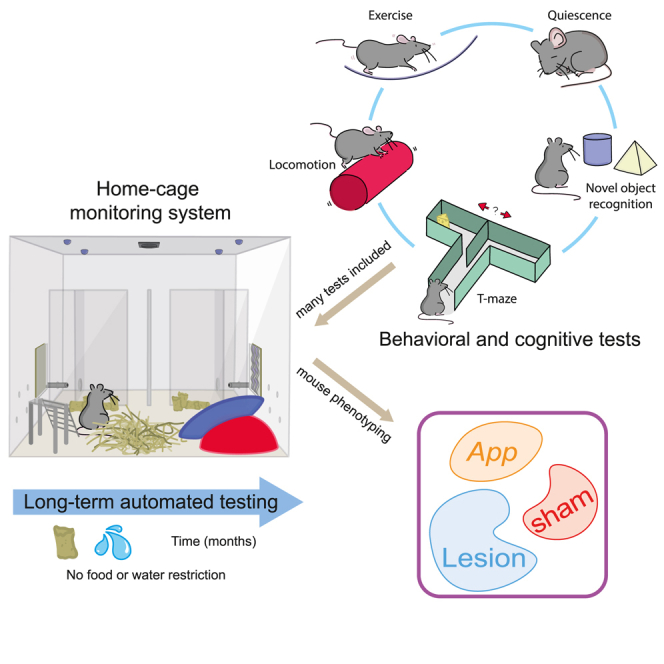

## Teaser

Ho et al. develop a home-cage system for mice with a fully automated T-maze, novel object recognition, and object-in-place tasks, as well as monitoring of locomotion. The system shows accuracy comparable to analogous standard tests and can be used for large-scale behavioral screening for genes and neural circuits underlying learning and memory.

## Introduction

Characterizing animals’ natural behaviors^1^^,^^2^ is paramount for understanding how the brain works. Currently, available tests are limited in scope and duration and often lack ethological relevance. In addition, the results commonly show significant variability due to variation in experimental conditions (e.g., food or water restriction, time of an experiment), individual animal differences, and potential subjective bias of the experimenter. To address these limitations, several automated platforms have been introduced.^3^^,^^4^^,^^5^^,^^6^ While progress in automated passive monitoring of motor behavior (e.g., an animal’s position, speed, posture) has been significant owing to the advances in software algorithms and hardware equipment, the so-called active phenotyping (whereby a mouse is required to perform memory and other cognitive tasks in their home cages) still presents a major challenge. Namely, all current commercial systems require elaborate pre-training involving food or water restriction.^7^^,^^8^^,^^9^^,^^10^ These requirements may induce biologically unnatural conditions and limit the duration for which the testing may be carried out. Lastly, the current systems are limited in the range of cognitive performances they are designed to test. Specifically, they implement different versions of place-preference tasks,^7^^,^^8^^,^^9^^,^^10^ whereby a mouse has to learn and relearn places associated with food or water rewards. No currently available home-cage-based monitoring system includes automated T-maze alternation,^11^^,^^12^ novel object,^13^^,^^14^ and object-in-place^15^ recognition tasks.

Here we describe the smart-Kage, a home-cage monitoring system for fully automated comprehensive cognitive and behavioral phenotyping of individually housed mice, compatible with long-term experiments. To demonstrate the usefulness of this system for basic and translational research, we characterized a small group of mice with hippocampal and medial entorhinal lesions known to exhibit substantial impairments on spatial memory tasks,^16^ as well as the widely used *App*^*NL-G-F*^ Alzheimer’s disease (AD) mouse model.^17^ Cognitive tasks include T-maze-like alternation (“smart T-maze”), novel object recognition (“smart NOR”), and object-in-place recognition (“smart OPR”) tasks carried out continuously and simultaneously in the smart-Kage without any interference from the experimenter. These tasks constitute some of the most widely used spatial memory tasks and are part of most standard behavioral test batteries designed to assess learning and memory.^18^ In addition, the system monitors an animal’s position, water consumption, quiescence, and locomotion patterns. We show that mice with hippocampal lesions can be separated from those with medial entorhinal lesions and sham controls on an individual animal basis. Moreover, in tandem with a short (∼7 days) test on a standard forced-choice T-maze task, individual mice from all three groups could be separated with high (>90%) accuracy. Finally, we could identify individual *App*^*NL-G-F*^ mice with 80% (4/5 mice) accuracy, which was comparable to the performance of the analogous gold-standard T-maze, NOR, and OPR tests.

## Results

### The smart-Kage system

**Figure 1:**
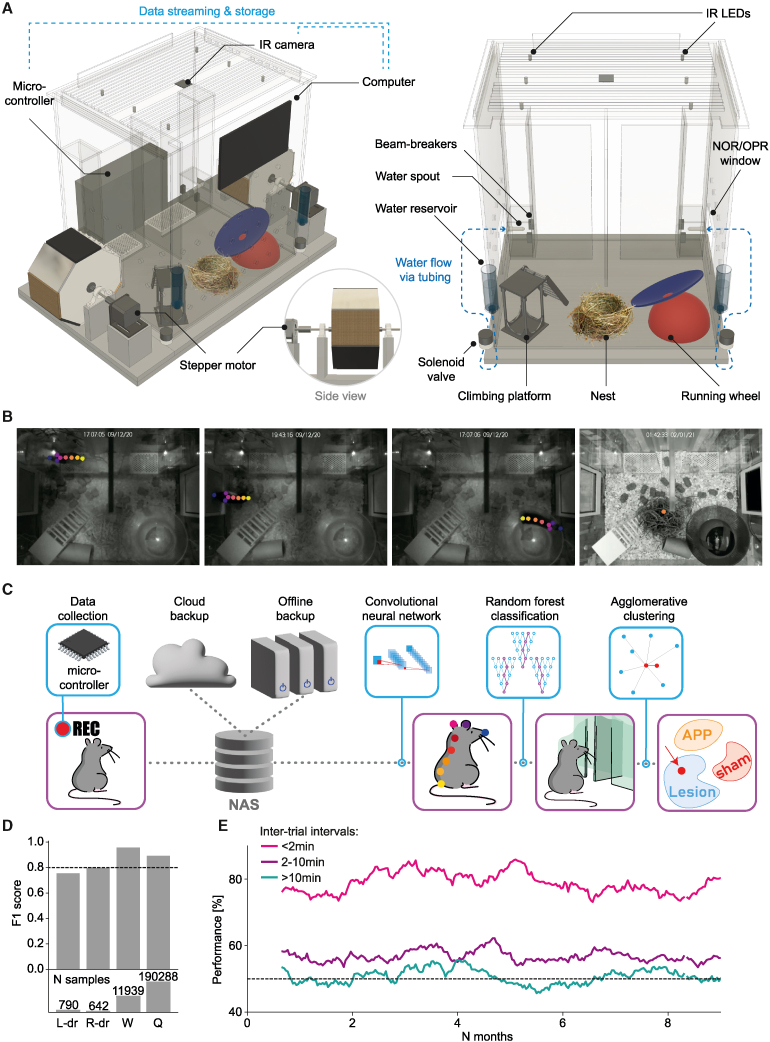
The smart-Kage system (A) Side (left) and front (right) views of the smart-Kage with all components labeled. The schematics is provided up to scale. (B) Top views of the smart-Kage interior with example CNN-based video tracking of different mouse behaviors. From left to right: mouse drinking, mouse exploring the surface of a side panel, mouse running on the wheel, and mouse in a quiescent state inside the nest. Each dot labels a specific mouse body part. (C) Automated phenotyping pipeline. Phenotyping begins by collecting top-view videos of the smart-Kage interior through an infrared (IR) camera. The data are stored on network-attached storage (NAS) devices and backed up to the cloud and offline external storage. The videos are then analyzed using CNN to obtain mouse trajectories and body postures. A random forest classifier is used to assign behavioral labels. In the final stage, behavioral parameters are used to predict the underlying mouse phenotype using agglomerative (hierarchical) clustering. (D) The smart-Kage behavioral labeling accuracy (top) is evaluated as the ability of the classifier to avoid false and find true positives (F1 score). The number of ground-truth frames used to calculate the phenotyping performance is shown at the bottom; drum exploration (L-dr, R-dr) is a much sparser behavior compared to running on the wheel (W) or quiescence states (Q). Each T-maze trial prediction was immediately checked against the ground-truth activation of IR sensors in the nose-poke ports, resulting in 100% accuracy in this category (not shown). (E) An example of the performance on the smart T-maze at different ITI collected from one mouse continuously tested over >8 months. See also Figures S1–S3 and Video S1.

The smart-Kage consists of three connected compartments (two corridors and an open space compartment) separated by three transparent boundaries (Figures 1A, 1B, and S1A). Each corridor leads to a water spout accessed through a nose-poke port with infrared sensors to detect the mouse’s drinking attempts. On each side of the smart-Kage, 20 mL water reservoirs are connected to the drinking spouts via small solenoid valves attached to the sides of the cage, which automatically open whenever a mouse triggers infrared sensors at the “correct” nose-poke ports.

The smart T-maze alternation task is designed to emulate the standard T-maze alternation task,^11^^,^^12^^,^^19^ which is a highly sensitive working memory test for detecting hippocampal damage^20^ because its successful execution requires an animal to remember its previous choices. Spontaneous alternation is a natural tendency of mature rodents to alternate their choice arms on a T-maze (or Y-maze), which becomes severely impaired in rodents with hippocampal lesions.^21^ The forced-choice alternation test is analogous to the spontaneous alternation test. However, in the former case the task consists of a sample and a test phase: during the sample phase, one of the choice arms is blocked by the experimenter, with the reward placed at the end of the freely accessible arm; during the test phase, the animal must choose the opposite arm from the previously visited one to receive the reward (Figure S1G). The impairment is negatively correlated with the duration between two consecutive choices called the inter-trial interval (ITI).^12^ Normally, standard spontaneous alternation, as well as forced-choice T-maze tasks, are conducted with a fixed ITI,^12^^,^^21^ which for practical considerations is usually constrained to <1–5 min (at longer intervals the trials become prohibitively long, and the mouse starts jumping out of the “start arm”). The smart T-maze task requires the mouse to alternate between the left and the right corridors to activate the water release, probing the mouse’s ability to recall the position of its previous choice (Figure S1G). The water is supplied for as long as the mouse keeps its nose in the port (short <1 s withdrawals were allowed before shifting the active spout to the opposite side). Similar to the ITI in standard T-maze tasks, ITI was measured as the time elapsed between different spout visits, i.e., the time between a mouse leaving the corridor after the nose poke and the next trigger of infrared sensors at a nose-poke port. The ITI is not enforced on the mouse (i.e., a mouse freely chooses when to make the next visit to a drinking spout) and, consequently, the performance of a whole range of ITIs (ranging from tens of seconds to a few hours) can be measured on the smart alternation task.

The smart NOR and OPR memory tasks take advantage of rodents’ innate tendency to exhibit increased exploratory activity toward new stimuli, similar to the analogous standard NOR and OPR memory tasks.^13^^,^^14^^,^^22^ Standard NOR and OPR tasks begin with a sample phase, whereby a mouse is placed in the familiar open arena and is presented with two unfamiliar objects that they can directly explore for several minutes (Figure S1H). After a fixed ITI ranging from seconds to days, the mouse is returned to the same familiar arena for a test phase and is presented with two objects, one familiar and one unfamiliar. In the OPR task, both objects are familiar during a test phase, but their locations are swapped. It has been suggested that the performance on standard NOR and OPR tasks may be affected in rodents with parahippocampal and hippocampal lesions, respectively.^22^ The smart-Kage incorporates an analogous NOR task, which is implemented by two rectangular panels (6.4 cm × 9.2 cm) positioned symmetrically on each side wall (Figures 1A and S1). The surface of the panels can change between eight distinctly different textures and colors (e.g., aluminum foil, different grades of sanding paper, plastic surfaces, etc.), and a mouse can directly explore them by touch, smell, and vision, similar to direct object exploration in standard NOR and OPR tasks. The surface panels are attached to two octagonal drums flanking the sides of the smart-Kage. Different surface panels are presented via the rotation of the drums. The drums are rotated by rotors placed in the centers of the drums (Figures 1A and S1). The rotation occurs only when a mouse is engaged with one of the water spouts, so it cannot directly observe the change. The drums are set to rotate once every two days around the middle of the dark phase (i.e., when the light in the mouse-holding room is off). The smart NOR task is quantified by measuring the exploration time associated with the change of the surface panel. The changes can happen on either wall individually (left or right NOR) or on both walls simultaneously (double NOR). The smart OPR task consists of presenting the same patterns as previously, but at “swapped” locations. We also implemented hybrid changes, with one of the walls assuming the pattern identical to the previous one on the opposite wall while the latter changes to a completely new unseen pattern or remains unchanged (Figure S1F).

Mouse activity within the smart-Kage was continuously recorded by an overhead infrared (IR) camera at two frames per second (0.5 s temporal resolution). The mouse’s precise position was determined by employing a deep convolutional neural network (CNN)^23^ with 1.85 mm spatial resolution (Figures 1A–1C and S2A–S2C; STAR Methods). Mouse behaviors were grouped into four distinct categories of interest (Figure 1B and Video S1): (1) T-maze choices; (2) drum-panel exploration; (3) running on the wheel; and (4) quiescence states. The behaviors of interest were inferred from the collected set of mouse trajectories and body postures using a random forest classifier (Figure 1C), which achieved >80% prediction accuracy when compared against manually annotated ground-truth frames (Figure 1D and STAR Methods). All behavioral and cognitive phenotyping tests were run automatically, continuously, and in parallel without any interference from the experimenter. Mice quickly learned the tasks without any need for water or food restrictions. Importantly, the system performance was stable over time and was well suited for long-term studies (Figure 1E; >8 months, limited only by the duration of the experiment).

Video S1. A collection of mouse behaviors in the smart-Kage with example CNN-based video tracking, related to Figure 1 5 distinct behaviors are shown: mouse drinking from the left water spout of the T-maze forced-choice alternation test (label: “T-maze”), mouse exploring the surface of the right side panel of the NOR/OPR test (label: “NOR/OPR”), mouse running on the wheel (label “Running wheel”), mouse sleeping inside the straw nest (label: “Quiescence”), and mouse displaying thigmotaxic behavior (label: “Thigmotaxis”). Each dot labels a specific mouse body part, as detected by the CNN.

### System application for phenotyping different mouse groups

To demonstrate the usefulness of the smart-Kage for cognitive and behavioral phenotyping, we first tested three groups of C57BL/6J mice with the experimenter blinded to the mouse’s phenotype: mice with ibotenic-acid-induced lesions in (1) the hippocampus (HP mice, n = 5); (2) medial entorhinal cortex (mEC mice, n = 4); and (3) a control group (control mice, n = 9) that received sham surgical procedures in the hippocampus, medial entorhinal cortex, or medial prefrontal cortex (Figure S3). All sham groups were combined into a single control group for further analysis, since there were no detectable behavioral or cognitive differences among them. The sample size of the HP group was chosen *a priori* based on the expected large effect size (Cohen’s d = ∼2.5) of the performance on the standard T-maze alternation task of HP vs. control mice.^24^^,^^25^ To achieve statistically robust conclusions, the estimated minimal group size of 4 is required with the power set to 0.8, the significance level to 5%, and the expected effect size of 2.5 (to calculate a sample size, we used the G∗Power software package with two-tailed Wilcoxon signed-rank test [one sample case]). The performance of mEC mice on similar tasks is less well characterized. As a result, we used the estimated sample size for the HP group (i.e., n = 4). All mice were randomly assigned to each group prior to commencing the study. The mice were run in two batches (Figure S1E and Table S1). They were tested for ∼1 month in the smart-Kages before lesioning, followed by an additional 2 months post-surgery testing.

To benchmark the performance of the smart-Kage, the same mice also underwent a battery of gold-standard spatial memory tests (T-maze forced-choice alternation, NOR, and OPR tasks)^8^^,^^11^^,^^13^^,^^14^^,^^26^ either after (the second batch) or both before and after (the first batch) they were tested in the smart-Kages (Figure S1E).

To demonstrate the usefulness of the smart-Kage for translational research, we also blindly tested a small number of *App*^*NL-G-F*^ mice (Table S1), which were previously reported to exhibit mild cognitive deficits on spatial memory tasks.^17^^,^^27^

Finally, we characterized ten additional C57BL/6J mice with no lesions (Table S1) to test the generality of our clustering analysis.

### Smart T-maze task

**Figure 2:**
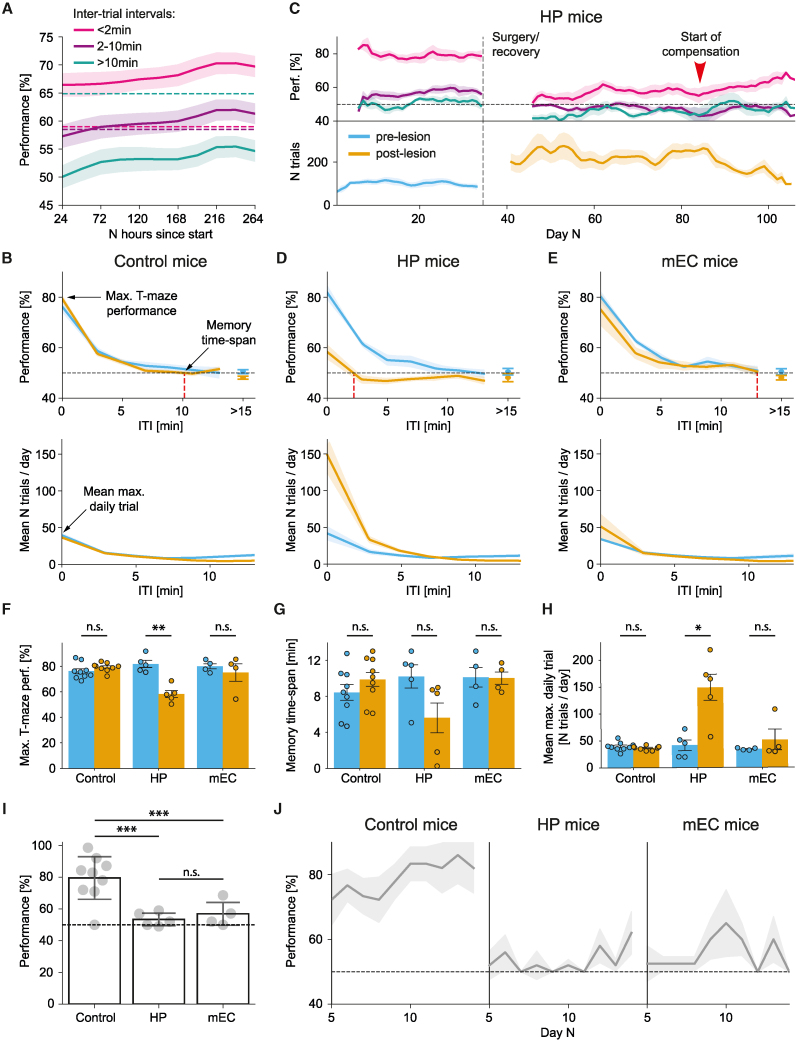
Smart T-maze alternation task (A) The smart T-maze alternation task is rapidly learned at shorter ITI periods (<10 min). Short ITI (<2 min): magenta; medium ITI (2–10 min): purple; long ITI (>10 min): teal. The performance on longer ITI periods (>10 min) is below chance levels (p < 0.05, binomial test). (B) The average distribution of performance (top) and daily frequency of spout visits (bottom) in control mice. The red dashed line indicates the memory time span (ITI at 50% T-maze performance). (C) Running average performance at different ITI intervals (top) and frequency of spout visits (bottom) in HP mice before and after the hippocampal lesions (vertical dashed line). The performance for all ITI domains significantly dropped after the lesion, accompanied by a significant increase in spout visits. The performance improved after ∼1.5 months post lesion (red arrowhead). The horizontal dashed line indicates chance-level performance. (D and E) The average distribution of performance (top) and daily frequency of spout visits (bottom) in HP (D) and mEC (E) mice, respectively. The red dashed line indicates the memory time span. (F–H) Maximum performance (F), memory time span (G), and maximum number of daily drinking attempts (H) in control, HP, and mEC mice pre-surgery (blue) and post-surgery (orange). (F) Maximum performance between pre-lesion vs. post-lesion is significantly reduced in HP mice but not in mEC or control groups (control: 76.3 ± 1.9% vs. 79.4 ± 1.1%, t = −1.5796, p = 0.2292; HP: 81.8 ± 2.6% vs. 58.3 ± 2.7%, t = 6.2765, p = 0.0099; mEC: 80.3 ± 2.0% vs. 75.1 ± 6.1% , t = 0.5452, p = 0.6235). (G) Memory time spans are not significantly different between pre- vs. post-lesion in all groups (control: 8.4 ± 0.8 min vs. 9.9 ± 0.8 min, t = −1.0364, p = 0.4954; HP: 10.2 ± 1.2 min vs. 5.6 ± 1.7 min, t = 2.6388, p = 0.1731; mEC: 10.1 ± 1.1 min vs. 10.0 ± 0.7 min, t = 0.0639, p = 0.9531). (H) Number of spout visits during pre- vs. post-lesion is significantly increased in HP mice while it remains comparable in mEC and control groups (control: 39.7 ± 2.5 visits/day vs. 36.8 ± 1.2 visits/day, t = 1.0214, p = 0.4122; HP: 41.7 ± 8.9 visits/day vs. 148.4 ± 24.0 visits/day, t = −5.8164, p = 0.0129; mEC: 33.9 ± 0.9 visits/day vs. 51.2 ± 16.4 visits/day, t = −0.9501, p = 0.4122). (I) The performance of the same mice on the standard T-maze task. The performances between control and test groups are significantly different (control vs. HP: t = 4.7058, p = 0.0008; control vs. mEC: t = 3.5735, p = 0.0046), whereas there is no significant difference between HP and mEC mice (t = −0.8242, p = 0.4370). (J) Daily average performance on a standard T-maze task in control (left), HP (middle), and mEC (right) mice, respectively. Pre-lesion period, blue; post-lesion period, orange. All data are represented as mean ± standard error of the mean (SEM). Independent-samples Student’s t test was used for all comparisons, unless stated otherwise. The normality of the data was verified with a Shapiro-Wilk test, and p values adjusted for false discovery rate with Benjamini-Hochberg correction. ∗p < 0.05, ∗∗p < 0.01, ∗∗∗p < 0.005; n.s., not significant. See also Figures S1G and S3 and Table S1.

Since access to the water spouts was unrestricted, ITIs were unconstrained. For visualization purposes, we have arranged these ITIs into three groups, <2 min, 2–10 min, and >10 min, reflecting short, mid-range, and long-term working memory, respectively. All pre-lesioned mice rapidly learned the smart T-maze task, performing above chance levels after 1 day at <2 min ITIs and after ∼2.5 days at 2–10 min ITIs (Figure 2A). The performance dropped rapidly with longer ITIs, reaching the chance level at ∼10 min ITI (Figure 2B), consistent with the working memory time span measured on the standard T-maze task.^12^ The spatial working memory was significantly impaired in mice with hippocampal lesions compared to their pre-lesion performance and sham controls (Figures 2C and 2D). Specifically, we observed a significant drop in maximum performance (Figures 2D and 2F: 81.8 ± 2.6% pre-lesion vs. 58.3 ± 2.7% post-lesion, t = 6.2765, p = 0.0099, paired Student’s t test, significant after Benjamini-Hochberg correction, p < 0.05) and a non-significant reduction in the working memory time span (Figures 2D and 2G: 10.2 ± 1.2 min pre-lesion vs. 5.6 ± 1.7 min post-lesion, t = 2.6388, p = 0.1731, paired Student’s t test). On the other hand, there was no significant change in maximal performance in mEC mice (Figures 2E–2F: 80.3 ± 2.0% pre-lesion vs. 75.1 ± 6.1% post-lesion, t = 0.5452, p = 0.6235, paired Student’s t test). Notably, in HP mice, the performance started to improve after ∼1.5 months post lesion (Figure 2C). This performance compensation timescale is comparable to the observations in other well-known hippocampal-dependent tests, such as the Morris water maze (∼43 days).^28^ We also found that impairment on the smart T-maze alternation task in HP mice was accompanied by a significant increase in spout visits at shorter ITIs (Figures 2D and 2H: 41.7 ± 8.9 visits/day pre-lesion vs. 148.4 ± 24.0 visits/day post-lesion, t = −5.8164, p = 0.0129, paired Student’s t test, significant after Benjamini-Hochberg correction, p < 0.05). The results are consistent with previous findings demonstrating larger water consumption in animals with hippocampal lesions.^29^ No such phenotype was observed in mice with entorhinal lesions (Figures 2E and 2H: 33.9 ± 0.9 visits/day pre-lesion vs. 51.2 ± 16.4 visits/day post-lesion, t = −0.9501, p = 0.4122, paired Student’s t test). Statistical tests for additionally tested T-maze behavioral features, which were not significantly different between groups, are shown in Figure S4A.

Importantly, unlike the difference in performance between the HP and mEC groups observed on the smart T-maze task, mice with both hippocampal and entorhinal damage showed dramatic impairments on a standard forced-choice alternation T-maze task (Figures 2I and 2J). Our findings indicate that although both standard and smart T-maze tasks are hippocampal dependent, they have remarkably different sensitivity to mEC lesions, which appears to depend on the lesion size (Figure S3). Currently the underlying cause of this difference remains unknown. However, this offers an unprecedented opportunity to use both tests in tandem to distinguish between the hippocampal, medial entorhinal, and control mice on an individual animal basis with 94% accuracy.

### Smart NOR and OPR tasks

**Figure 3:**
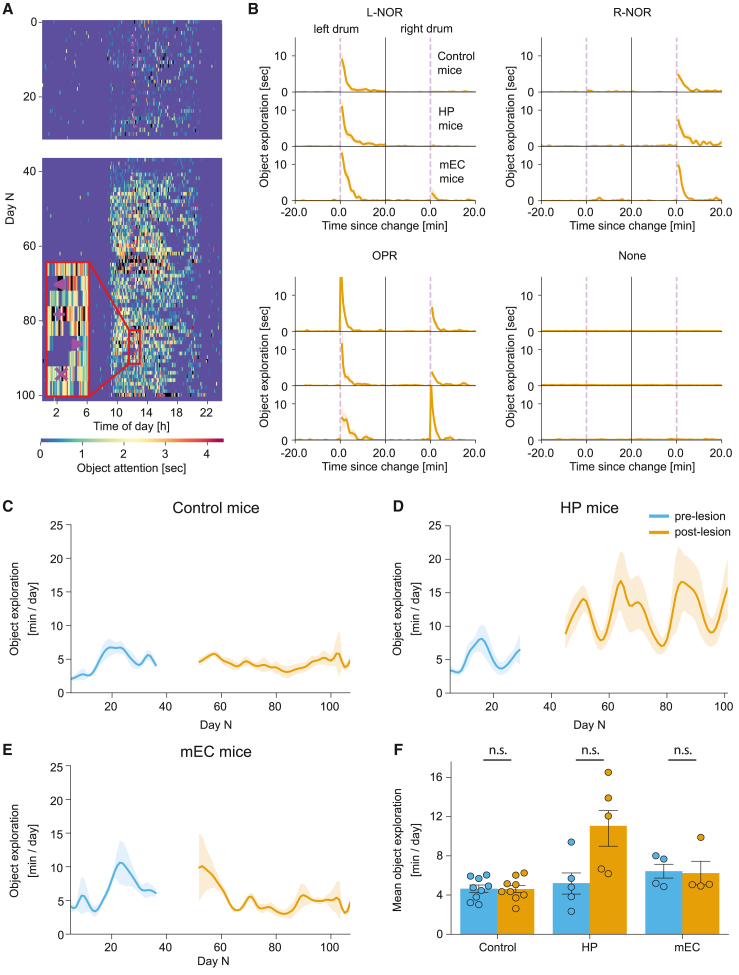
Smart novel object and object-in-place recognition tasks (A) A typical ethogram showing the exploration of side panels of a single HP mouse tested for ∼3 months. The time of lesion surgery and the following week of recovery is indicated by the white gap between days 30 and 40. Purple shapes indicate the time of side-panel change (left-facing triangle, left drum NOR; plus sign, left drum OPR + right drum NOR; right-facing triangle, right drum NOR; ×, left drum NOR + right drum OPR). (B) The average mouse response to L-NOR (top left), R-NOR (top right), and OPR (bottom left) tasks 20 min before and after the change of a side panel, indicated by the purple dashed line. Top, middle, and bottom rows of each plot correspond to control, HP, and mEC mice, respectively. The left and right columns of each plot correspond to the exploration time of the left and the right side panel, respectively. The bottom left and bottom right plots show animal exploration time when both or none of the side panels changed. The exploration on days with no change was measured at 12:00 p.m. for direct comparison. (C–E) Average daily side-panel exploration time for control (C), HP (D), and mEC (E) mice, respectively. (F) Mean object exploration time between pre-lesion vs. post-lesion remains unchanged in all three groups (control: 4.6 ± 0.4 min/day vs. 4.6 ± 0.4 min/day, t = 0.0541, p = 0.9582; HP: 5.2 ± 1.1 min/day vs. 11.1 ± 1.8 min/day, t = −2.291, p = 0.2514; mEC: 6.4 ± 0.7 min/day vs. 6.2 ± 1.1 min/day, w = 4, p = 0.9102 (Wilcoxon signed-rank test was used for mEC). Pre-lesion period, blue; post-lesion period, orange. All data are represented as mean ± standard error of the mean (SEM). Independent-samples Student’s t test was used for all comparisons, unless stated otherwise. The normality of the data was verified with a Shapiro-Wilk test, and p values adjusted for false discovery rate with Benjamini-Hochberg correction. ∗p < 0.05, ∗∗p < 0.01, ∗∗∗p < 0.005; n.s., not significant. See also Figures S1F, S1H, and S5.

Our results show that all groups increased their exploration time in response to the change of a surface panel (Figures 3A and 3B). Similar to observations in standard tests, the increase in exploration was a natural behavior that did not require any pre-training and occurred from the first encounter (Figures S5A–S5C). The time to notice the change was comparable in all mouse groups (Figure 3B; 1.2 ± 0.1 min vs. 1.6 ± 0.2 min vs. 1.4 ± 0.2 min in control, HP, and mEC groups, respectively; F = 2.58, p = 0.109, one-way ANOVA). There was also no significant change in average exploration time pre- and post-lesion in both smart NOR and OPR tasks in any of the different mouse groups (Figures 3C–3F; p > 0.25). Similarly, we found no significant difference between all groups when tested on the standard NOR and OPR tasks (Figures S5D and S5E; STAR Methods).

### Locomotion and quiescent behaviors

In addition to measuring the performance of the mice on cognitive memory tasks, we also assessed their locomotion and quiescence patterns. Voluntary activity on the running wheel has been previously associated with increased hippocampal neurogenesis and improvements in mouse’s performance on spatial memory tasks.^30^^,^^31^ However, it is unknown how mice with hippocampal and medial entorhinal damage use a running wheel.

**Figure 4:**
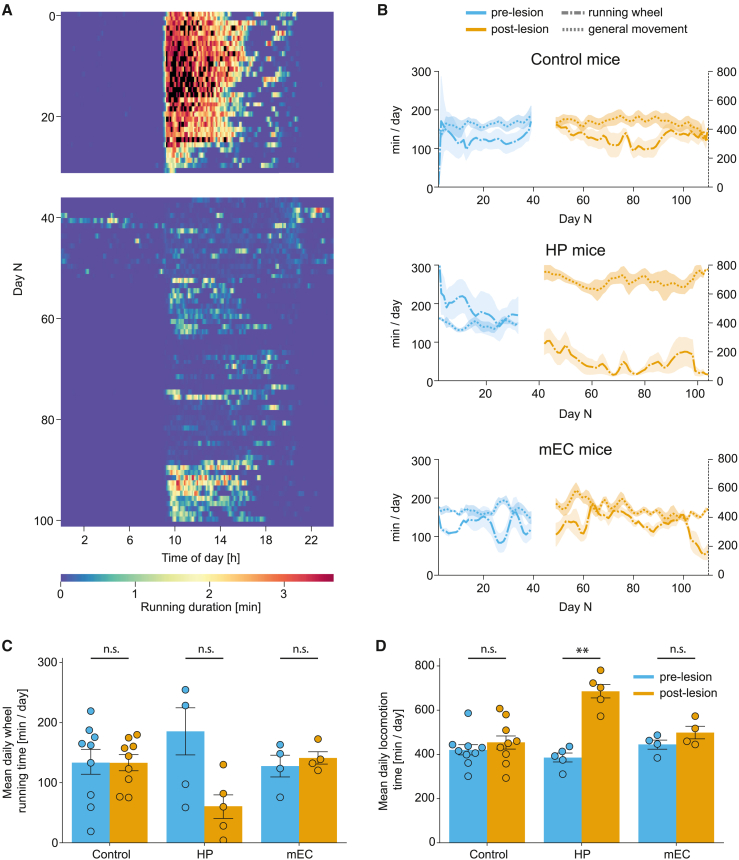
Running on the wheel and general locomotion in the smart-Kage (A) A typical ethogram showing daily wheel-running activity of a single HP mouse tested for ∼3 months. The time of lesion surgery and the following week of recovery is indicated by the white gap between days 30 and 40. (B) Average general locomotion (dotted) and wheel-running (dashed-dotted) behaviors in control (top), HP (middle), and mEC (bottom) mice, respectively. (C) Average time per day spent running on the wheel is unchanged between pre- vs. post-lesion in all groups (control: 133.4 ± 20.9 min/day vs. 133.1 ± 12.5 min/day, t = −0.0242, p = 0.9813; HP: 185.5 ± 40.5 min/day vs. 60.9 ± 19.5 min/day, t = 2.2154, p = 0.2733; mEC: 127.7 ± 16.6 min/day vs. 141.1 ± 9.7 min/day, t = −0.6588, p = 0.8357). (D) HP mice were significantly more mobile after lesion while mEC and control mice displayed no change in overall movement between pre- vs. post-lesion (control: 420.2 ± 24.1 min/day vs. 454.6 ± 31.6 min/day, t = −1.0067, p = 0.3435; HP: 385.5 ± 19.1 min/day vs. 685.3 ± 31.5 min/day, t = −6.7665, p = 0.0075; mEC: 445.1 ± 19.0 min/day vs. 498.6 ± 25.4 min/day, t = −1.1974, p = 0.3435). Pre-lesion period, blue; post-lesion period, orange. All data are represented as mean ± standard error of the mean (SEM). Independent-samples Student’s t test was used for all comparisons, unless stated otherwise. The normality of the data was verified with a Shapiro-Wilk test, and p values adjusted for false discovery rate with Benjamini-Hochberg correction. ∗p < 0.05, ∗∗p < 0.01, ∗∗∗p < 0.005; n.s., not significant.

Our results show that mice with hippocampal damage did not show a statistically significant change in time spent running on the wheel (Figures 4A–4C: 185.5 ± 40.5 min/day pre-lesion vs. 60.9 ± 19.5 min/day post-lesion, t = 2.2154, p = 0.2733, paired Student’s t test) in contrast to a significant ∼78% increase in their overall movement in the smart-Kage (Figures 4B and 4D: 385.5 ± 19.1 min/day pre-lesion vs. 685.3 ± 31.5 min/day post-lesion, t = −6.7665, p = 0.0075, paired Student’s t test, significant after Benjamini-Hochberg correction, p < 0.05). Interestingly, the movement increase was accompanied by instances of stereotypical behaviors, such as running in a circular trajectory that was not observed in control mice (Video S1). No change in running on the wheel was observed in mice with medial entorhinal lesions (Figures 4B and 4C: 127.7 ± 16.6 min/day pre-lesion vs. 141.1 ± 9.7 min/day post-lesion, t = −0.6588, p = 0.8357, paired Student’s t test) or the control group (Figures 4B and 4C: 133.4 ± 20.9 min/day pre-lesion vs. 133.1 ± 12.5 min/day post-lesion, t = −0.0242, p = 0.9813, paired Student’s t test). These groups also did not exhibit any noticeable change in the overall movement in the smart-Kage post-surgery (Figures 4B and 4D: mEC mice: 445.1 ± 19.0 min/day pre-lesion vs. 498.6 ± 25.4 min/day post-lesion, t = −1.1974, p = 0.3435, paired Student’s t test; control mice: 420.2 ± 24.1 min/day pre-lesion vs. 454.6 ± 31.6 min/day post-lesion, t = −1.0067, p = 0.3435, paired Student’s t test).

**Figure 5:**
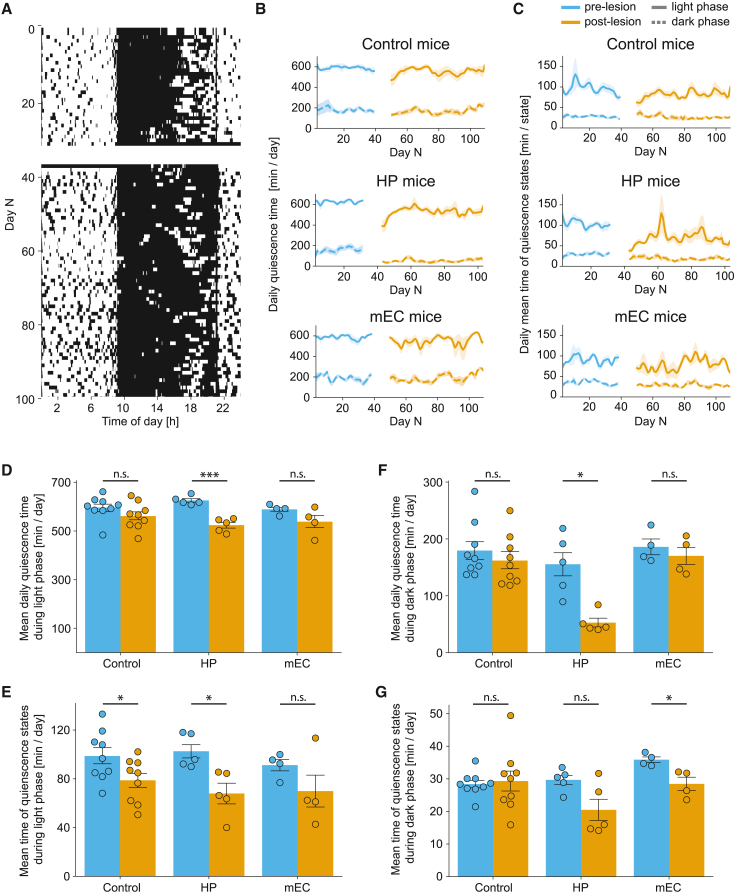
Quiescence states in the smart-Kage (A) A typical ethogram showing quiescence states of the same mouse as in Figure 4A. White and black regions indicate quiescence and mobile intervals, respectively. The time of lesion surgery and the following week of recovery is marked as the white gap between days 30 and 40. (B and C) The total daily average time spent in quiescence (B) and average duration of each quiescent state (C) in control (top), HP (middle), and mEC (bottom) mice. Solid and dashed lines correspond to light and dark phases, respectively. (D–G) Mean daily quiescence time and the mean duration of a single quiescent state during the light (D and E) and dark (F and G) phases in control, HP, and mEC mice, respectively. (D) Mean daily quiescence time during light phase (control: 596.1 ± 15.3 min/day vs. 561.3 ± 17.2 min/day, t = 2.2325, p = 0.0842; HP: 625.4 ± 7.1 min/day vs. 524.1 ± 11.1 min/day , t = 10.630, p = 0.0012; mEC: 588.8 ± 8.7 min/day vs. 538.2 ± 24.8 min/day, t = 1.3289, p = 0.2759). (E) Mean duration of quiescent state during light phase (control: 98.7 ± 6.5 min/day vs. 78.8 ± 5.6 min/day, t = 3.4773, p = 0.0252; HP: 102.6 ± 5.5 min/day vs. 68.0 ± 7.4 min/day, t = 3.6773, p = 0.032; mEC: 91.2 ± 4.3 min/day vs. 69.9 ± 13 min/day, t = 1.418, p = 0.2512). (F) Mean daily quiescence time during dark phase (control: 179.5 ± 15.3 min/day vs. 161.9 ± 13.9 min/day , t = 1.2955, p = 0.3168; HP: 155.4 ± 21.0 min/day vs. 52.6 ± 7.2 min/day, t = 4.4055, p = 0.0348; mEC: 186.0 ± 12.1 min/day vs. 170.1 ± 14.1 min/day, t = 1.1984, p = 0.3168). (G) Mean duration of quiescent state during dark phase (control: 28.4 ± 1.2 min/day vs. 29.3 ± 3.0 min/day, t = 0.2471, p = 0.811; HP: 29.7 ± 1.4 min/day vs. 20.5 ± 3.2 min/day, t = 2.4045, p = 0.111; mEC: 35.9 ± 0.8 min/day vs. 28.5 ± 1.8 min/day, t = 5.9352, p = 0.0288). Pre-lesion period, blue; post-lesion period, orange. All data are represented as mean ± standard error of the mean (SEM). Independent-samples Student’s t test was used for all comparisons, unless stated otherwise. The normality of the data was verified with a Shapiro-Wilk test, and p values adjusted for false discovery rate with Benjamini-Hochberg correction. ∗p < 0.05, ∗∗p < 0.01, ∗∗∗p < 0.005; n.s., not significant.

Next, we assessed quiescent states of the mice, defined as periods when the mouse was completely and continuously motionless for at least 5 min (Figure 5A and STAR Methods). We found that control mice spent most (78%–83%) of their 12-h light phase immobile or sleeping (Figures 5B–5D: 596.1 ± 15.3 min/day pre-lesion vs. 561.3 ± 17.2 min/day post-lesion, t = 2.2325, p = 0.0842, paired Student’s t test) with occasional brief activity periods usually related to water or food consumption. On the other hand, as expected, they spent only around 23%–25% of the time immobile during the dark phase (Figures 5B and 5F: 179.5 ± 15.3 min/day pre-lesion vs. 161.9 ± 13.9 min/day post-lesion, t = 1.2955, p = 0.3168, paired Student’s t test). Mice with hippocampal lesions showed significantly disrupted and less regular quiescence patterns (Figures 5A–5C) accompanied by an overall significant decrease in average immobility time during both light and dark phases (Figures 5A, 5B, 5D, and 5F: light phase: 625.4 ± 7.1 min/day pre-lesion vs. 524.1 ± 11.1 min/day post-lesion, t = 10.630, p = 0.0012; dark phase: 155.4 ± 21.0 min/day pre-lesion vs. 52.6 ± 7.2 min/day post-lesion, t = 4.4055, p = 0.0348, paired Student’s t test, significant after Benjamini-Hochberg correction, p < 0.05). Notably, both hippocampal (102.6 ± 5.5 min/day pre-lesion vs. 68.0 ± 7.4 min/day post-lesion, t = 3.6773, p = 0.032, paired Student’s t test, significant after Benjamini-Hochberg correction, p < 0.05) and control (98.7 ± 6.5 min/day pre-lesion vs. 78.8 ± 5.6 min/day post-lesion, t = 3.4773, p = 0.0252, paired Student’s t test, significant after Benjamini-Hochberg correction, p < 0.05) groups showed a significant decrease in the average “nap” duration during the light phase, while mEC mice showed similar non-significant trends (91.2 ± 4.3 min/day pre-lesion vs. 69.9 ± 13 min/day post-lesion, t = 1.418, p = 0.2512, paired Student’s t test), likely reflecting some general age-related (or, alternatively, experience in the smart-Kage related) trend (Figure 5E).

Finally, the mice with medial entorhinal lesions showed a significant decrease in average “nap” duration (an average duration of a single immobility state) during the dark phase (Figure 5G: 35.9 ± 0.8 min pre-lesion vs. 28.5 ± 1.8 min post-lesion, t = 5.9352, p = 0.0288, paired Student’s t test, significant after Benjamini-Hochberg correction, p < 0.05) while it was not significantly changed in other mouse groups. Additional behavioral features related to quiescence that showed no statistically significant changes are shown in Figures S4B and S4C.

### Automated classification of different mouse groups

**Figure 6:**
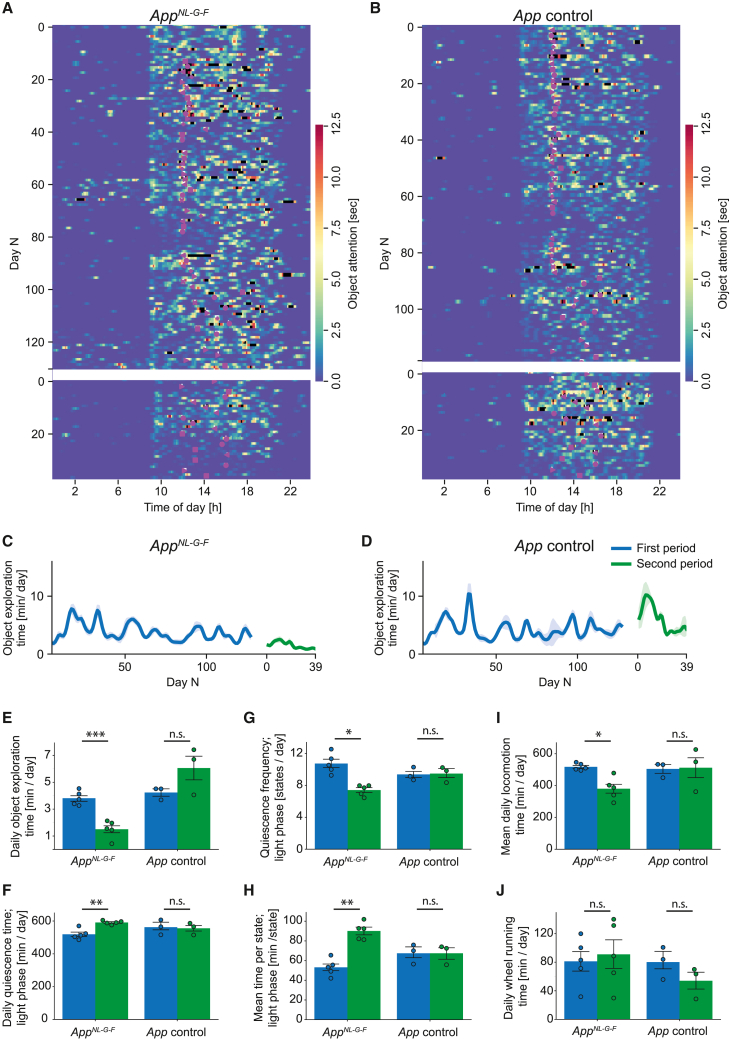
Phenotyping of *App*^*NL-G-F*^ mice in the smart-Kage (A and B) A typical ethogram showing the exploration of side panels of a single *App*^*NL-G-F*^ (A) and an age-matched control (B) mouse. The first testing period was over 4 months and the second period lasted ∼1.5 months. Between the end of the first and the start of the second period, there was an 8 month gap (white bar). Purple shapes indicate the time of side-panel changes (left-facing triangle, left drum NOR; plus sign, left drum OPR + right drum NOR; right-facing triangle, right drum NOR; ×, left drum NOR + right drum OPR). (C and D) Average daily side-panel exploration time for *App*^*NL-G-F*^ (C) and age-matched control (D) mice, respectively. (E–J) Average daily object exploration time (E), total quiescence time during light phase (F), the corresponding number of quiescent states per day, called quiescence frequency (G), and their average duration (H), locomotion (I), and running on the wheel (J) in *App*^*NL-G-F*^ and age-matched control mice between first vs. second period. (E) Average daily object exploration time (*App*^*NL-G-F*^: 3.8 ± 0.2 min/day vs. 1.5 ± 0.3 min/day, t = 7.7614, p = 0.003; control: 4.2 ± 0.2 min/day vs. 6.1 ± 0.8 min/day, w = 1, p = 0.8131 [Wilcoxon signed-rank test]). (F) Total quiescence time during light phase (*App*^*NL-G-F*^: 519.3 ± 12.6 min/day vs. 591.2 ± 4.3 min/day, t = −5.8559, p = 0.0084; control: 562.6 ± 21.4 min/day vs. 555.7 ± 17.3 min/day, t = 0.3743, p = 0.7441). (G) Quiescence frequency during light phase (*App*^*NL-G-F*^: 10.7 ± 0.5 states/day vs. 7.4 ± 0.3 states/day, t = 3.8942, p = 0.0352; control: 9.4 ± 0.4 states/day vs. 9.5 ± 0.5 states/day, t = 0.1381, p = 0.9028). (H) Mean duration of quiescence states during light phase (*App*^*NL-G-F*^: 53.1 ± 3.4 states/day vs. 90.1 ± 3.4 states/day, t = −5.6729, p = 0.0096; control: 67.5 ± 4.7 states/day vs. 67.4 ± 4.8 states/day, t = 0.0153, p = 0.9892). (I) Mean daily duration of general movment (*App*^*NL-G-F*^: 517.7 ± 8.7 min/day vs. 380.2 ± 28.0 min/day, t = 5.4728, p = 0.0108; control: 504.8 ± 22.6 min/day vs. 512.3 ± 64.2 min/day, t = −0.0837, p = 0.9409). (J) Daily wheel running time (*App*^*NL-G-F*^: 81.2 ± 13.3 min/day vs. 91.0 ± 18.4 min/day, t = -0.5654, p = 0.602; control: 80.2 ± 10.7 min/day vs. 54.0 ± 10.5 min/day, t = 1.4248, p = 0.5806). 5–9 months of age, blue; 18–20 months of age, green. All data are represented as mean ± standard error of the mean (SEM). Independent-samples Student’s t test was used for all comparisons, unless stated otherwise. The normality of the data was verified with a Shapiro-Wilk test, and p values adjusted for false discovery rate with Benjamini-Hochberg correction. ∗p < 0.05, ∗∗p < 0.01, ∗∗∗p < 0.005; n.s., not significant. See also Figures S6–S8 and Table S1.

Can we use the smart-Kage to achieve high sensitivity in classifying different mouse groups? To address this question, we blindly tested four different mouse groups comprised of already described HP, mEC, and control groups, and a newly added *App*^*NL-G-F*^ AD mouse model group and its age-matched controls (Figures 6, S4D–S4J, and S6–S8). *App*^*NL-G-F*^ mice and their controls were characterized at two different periods (Table S1) for 4 months (the first period) and 1.5 months (the second period), which were carried out 12.5 months apart to investigate how their behavior in the smart-Kage changes with time. We found that *App*^*NL-G-F*^ mice significantly decreased novel object exploration time (Figures 6A–6E: 3.8 ± 0.2 min/day vs. 1.5 ± 0.3 min/day during the first and the second period, respectively, t = 7.7614, p = 0.003, paired Student’s t test, significant after Benjamini-Hochberg correction, p < 0.05). They also remained immobile for longer, with longer average “nap” durations (Figures 6F–6H: average total time of immobility during the light cycle: 519.3 ± 12.6 min/day vs. 591.2 ± 4.3 min/day during the first and the second period, respectively, t = −5.8559, p = 0.0084, paired Student’s t test, significant after Benjamini-Hochberg correction, p < 0.05; and 53.1 ± 3.4 min/state vs. 90.1 ± 3.4 min/state during the first and the second period, respectively, t = 0.0048, p = 0.0096, paired Student’s t test, significant after Benjamini-Hochberg correction, p < 0.05). These changes were accompanied by lower general locomotion activity (Figure 6I: 517.7 ± 8.7 min/day vs. 380.2 ± 28.0 min/day, t = 5.4728, p = 0.0108, paired Student’s t test, significant after Benjamini-Hochberg correction, p < 0.05); however, on average no change in the time spent running on the wheel was observed (Figure 6J: 81.2 ± 13.3 min/day vs. 91.0 ± 18.4 min/day, t = −0.5654, p = 0.602, paired Student’s t test).

Next, we used 32 commonly applied cognitive and behavioral measurements to investigate whether mice can be accurately assigned to their corresponding groups. The measurements were based on one of the four categories described above (Figure S9 and Table S2): (1) the performance on the smart T-maze alternation task, (2) NOR and OPR tasks; (3) quiescence states; and (4) wheel-running behavior.

Of note, the measures within the category showed some degree of correlation; however, there was little to no correlation between the measurements from different categories (Figure S10 and STAR Methods). Significant correlations between each type of behavior were identified as those whose absolute value was higher than a threshold value calculated as the 95^th^ percentile value of randomly shuffling existing features across mice within each feature type (STAR Methods).

**Figure 7:**
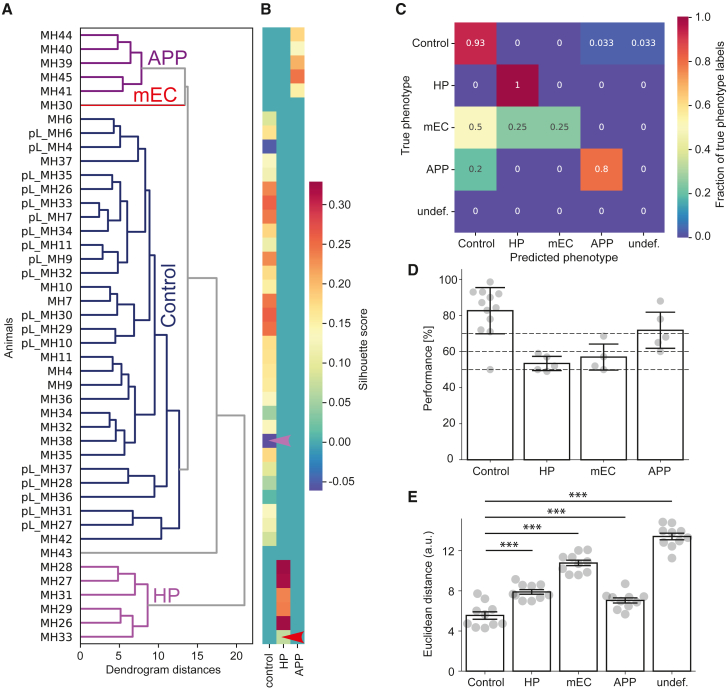
Behavioral clustering on an individual animal basis (A) Hierarchical clustering dendrogram of the 32-dimensional feature space. (B) Clustering silhouette plot. Warmer colors indicate good separation from neighboring clusters, whereas colder colors indicate potential outliers in respective clusters. An mEC outlier in the HP cluster and an *App*^*NL-G-F*^ outlier in the control cluster are marked with red and purple arrowheads, respectively. (C) Confusion matrix comparing actual and predicted animal identities detected by smart-Kage. The values indicate the percentage of animals assigned to each class. (D) Animal clustering based on the standard T-maze task. The dashed lines demarcate distinct clusters: controls at >70% performance, HP and mEC mice at 50%–60% performance, and *App*^*NL-G-F*^ mice at 60%–70% performance (control: 82.7 ± 13.4%; HP: 53.4 ± 4.4%; mEC: 56.9 ± 8.3%; *App*^*NL-G-F*^: 71.8 ± 11.2%). (E) The Euclidean distance between the newly added control mice and previously identified groups in behavioral feature space(control vs. HP: 5.5 ± 1.2 vs. 7.9 ± 0.8 [t = −5.18931, p = 8.232351e-05]; control vs. mEC: 5.5 ± 1.2 vs. 10.8 ± 0.9 [t = −10.75881, p = 5.729525e-09]; control vs. *App*^*NL-G-F*^: 5.5 ± 1.2 vs. 7.0 ± 0.8 [t = −3.20276, p = 4.932517e-03]; control vs. undef.: 5.5 ± 1.2 vs. 13.4 ± 1.1 [t = −15.21472, p = 4.063801e-11]). All data are represented as mean ± standard error of the mean (SEM). Independent-samples Student’s t test was used for all comparisons, unless stated otherwise. The normality of the data was verified with a Shapiro-Wilk test, and p values adjusted for false discovery rate with Benjamini-Hochberg correction. ∗p < 0.05, ∗∗p < 0.01, ∗∗∗p < 0.005; n.s., not significant. See also Figures S3 and S9 and Table S2.

The mice were grouped using an agglomerative (hierarchical) clustering algorithm to predict the underlying mouse phenotype (Figures 7A and 7B; STAR Methods). Specifically, we ran 25,000 optimization simulations, whereby in every simulation we tested a different combination of a clustering algorithm and its associated hyperparameters. The following common clustering algorithms were tested: k-means, Bayesian Gaussian mixture models, agglomerative clustering, OPTICS, spectral clustering, and affinity propagation. We chose a subset of clusterings that identified the only *a priori* known group (the control mice) with 100% accuracy. The final clustering was chosen from this subset after unblinding the remaining group identities (control, HP, mEC, and *App*^*NL-G-F*^mice). We found that agglomerative clustering using ward linkage and Euclidean distance metric showed the highest accuracy in identifying these groups. The accuracy was measured as the percentage of correctly identified mice (Figure 7C). It should be noted that no additional clustering simulations were run after unblinding, i.e., the optimal clustering was chosen from among simulations done before unblinding to minimize human bias. Next, we tested the clusters’ quality and stability by applying a leave-one-animal-out approach. We ran 10,000 clustering simulations, whereby one randomly chosen mouse was removed from the dataset and the same clustering was repeated on the remaining dataset. The resultant clusterings were compared with the original clustering using the mean Silhouette score^32^^,^^33^ and the chance-adjusted Rand Index (RI) score^34^^,^^35^ to quantify the quality and stability, respectively (STAR Methods). The estimated mean Silhouette score and RI value of our clustering were (mean ± SD) 0.1547 ± 0.0928 and 0.8926 ± 0.1463, respectively, which were significantly higher compared to chance threshold values calculated as the 95^th^ percentile values of the surrogate data generated by randomly shuffling existing features across mice within each feature type (STAR Methods), indicating an appreciable deviation of our original clustering from randomness.

Using this approach, we were able to correctly classify 100% of animals belonging to the control cluster (27/27; 18 pre-lesioned mice and 9 post-lesion sham control mice), 100% (5/5) of mice with hippocampal lesions, and 25% (1/4) of mice with medial entorhinal lesions (Figures 7A–7C). Interestingly, one of the mEC mice was clustered together with the HP group but stood out as an outlier within this group (Figure 7B, red arrowhead). The two remaining mice with medial entorhinal lesions were misclassified as controls. After unblinding the histology results, we found that the correctly identified mEC mouse had the largest volume of the medial entorhinal cortex removed, followed by the mouse within the HP cluster (Figure S3C). The mEC mice grouped with the controls had much more limited mEC lesions.

We also successfully identified 80% (4/5) of *App*^*NL-G-F*^ mice. The only misassigned *App*^*NL-G-F*^ mouse was classified as a control, although it was a strong outlier within the control group (Figure 7B, purple arrowhead). Of note, *App*^*NL-G-F*^ control mice were classified outside the general control cluster, likely due to their different genotype and/or age compared to pre-lesioned mice and sham controls (STAR Methods). The classification accuracy achieved by the smart-Kage was comparable to that of the standard T-maze, NOR, and OPR tasks (Figures 7C and 7D; 60% [3/5] *App*^*NL-G-F*^ mice). Thus overall, 7 (4/5 *App*^*NL-G-F*^ and 3/3 age-matched controls) out of 8 correct performances is significantly better than what would be expected by chance (p < 0.05, binomial test). In the case of the standard tests, the grouping was based on the performance on the T-maze alone, as results from NOR and OPR tasks did not serve as good predictors (Figure S5F and STAR Methods). Adding more tests to the battery, such as open-field exploration, could improve the accuracy of standard tests. However, each new test would require separate pre-training; currently, no standard test can separate HP and mEC mice.

Finally, to further demonstrate the analytical power of our approach, we tested an additional batch of ten new unlesioned C57BL/6J mice of the same sex (males) and age (16 weeks) as other pre-lesioned C57BL/6J mice, characterized over 30 days in the smart-Kages, to investigate which cluster they would be assigned to based on their proximity to our previously identified clusters. We found that all ten mice were closest to the control cluster (Figure 7E). This is further proof of principle of the robustness of our current classification approach.

## Discussion

Here we described a home-cage monitoring system (the smart-Kage) which incorporates T-maze alternation and NOR and OPR tests, enabling their fully automated repetitive execution over long periods. In the current study the phenotyping was focused on cognitive domains, especially important for research on neurological disorders. In line with previous studies using analogous standard tests, we showed that: (1) the span of the working memory in mice is ∼10 min^12^; (2) changes of external drum patterns in the smart-Kage resulted in their increased exploration in line with the standard NOR; (3) the swap of the two familiar patterns results in their increased exploration in line with the standard OPR task; (4) mice show a strong preference to run on the running wheel, in line with the previous observations^36^; and (5) locomotion activity of mice is strongly controlled by the circadian rhythm. Contrary to the standard T-maze alternation task, we were not required to implement any water or food restrictions. We showed that the delay in water consumption encountered after choosing an incorrect water spout was a sufficiently negative reinforcer to trigger rapid learning on the smart T-maze alternation task, eliminating any need for food or water restriction and thus contributing to the ethological relevance of our system. In addition, the smart-Kage also simultaneously characterizes a range of other non-cognitive behaviors, such as locomotion and quiescence states, which in the majority of cases likely serve as a proxy for sleeping patterns in mice.^37^

In the proof-of-principle experiments using small samples of mice with hippocampal, medial entorhinal, and sham lesions as well as the *App*^*NL-G-F*^ AD mouse model and their controls, we demonstrated the effectiveness of the smart-Kage by focusing on hippocampal-parahippocampal-dependent spatial working memory, novel object, and OPR behaviors. In a blind test, we showed that using the smart-Kage we could identify different groups of mice with high accuracy and sensitivity without making any assumptions about specific unambiguous group phenotypes. Instead, we relied solely on combining multiple behavioral measures recorded in the smart-Kage, which on their own often showed only non-significant behavioral and cognitive trends (at least in the small samples that we employed). Unlike previously reported approaches that used the “leave-one-animal-out procedure” (i.e., the identities of all but one animal were provided for cluster assignment), our clustering algorithm was trained only on the pre-lesioned mice representing a known control group. All other clusters, which included both lesion and sham post-lesion groups and mice with AD-associated genetic modifications, were produced automatically in a completely unsupervised way, and the best solution was chosen based on the optimal group assignment for all groups combined. We showed that our approach yielded results comparable to behavioral phenotyping using the three most prominent analogous standard memory tests. Importantly, newly added ten unlesioned C57BL/6J mice were automatically assigned to the “control cluster” based on their closest proximity in the multi-dimensional feature space. Thus, we expect that our home-cage monitoring system will provide a promising tool for automated phenotyping of more natural behaviors, probing spatial memory and object recognition, which may enable direct comparison across labs and improved standardization.

### Limitations of the study

The number of mice per group used in this study is limited except for a control group. The small number was afforded because of the strong effect size in mice with hippocampal lesions; however, larger groups may be useful for mice with mEC lesions and AD mice. Additionally, all experiments have been carried out in the same animal facility.

The training data for neural network (NN) models for tracking cannot be made publicly available, since it is a proprietary dataset. However, this will not prevent replicating the system, as it is a “helpful” but not necessary part of the system implementation. Creating training datasets is a standard part of the DeepLabCut algorithm,^23^ and they are chosen arbitrarily. Thus, each user can define their own training datasets from the raw image data.

The NOR and OPR tests were carried out only with short ITIs (up to a few minutes). Longer ITIs must be explored to identify values for which mice with hippocampal and mEC lesions and AD mice become impaired as predicted based on the observation in analogous standard tests.

Finally, the home-cage monitoring system is designed for single-mouse testing, which may be suboptimal in terms of potential stress induced by isolation and low throughput. If permitted by experimental design, the ability to test group-housed mice would increase throughput and may be more ethologically relevant. On the other hand, group housing may not be appropriate for some experiments as it will introduce noise associated with “crowd behavior” or with emerging hierarchical structures. Group housing also has another well-known issue that co-housed males tend to fight, which may inflict severe injuries and cause additional stress.

## STAR★Methods

### Key resources table

**Table:** 

REAGENT or RESOURCE	SOURCE	IDENTIFIER
**Chemicals**, **peptides**, **and recombinant proteins**		

Ibotenic acid	Sigma-Aldrich	I2765; CAS: 2552-55-8

**Deposited data**		

Mouse processed behavioral data	This paper	Zenodo: https://doi.org/10.5281/zenodo.8003569

**Experimental models**: **Organisms/strains**		

Mouse: C57BL/6J	Charles River	Strain Code 632
Mouse: App^NL−G-F^: Apptm3.1Tcs/Apptm3.1Tcs	Mary Lyon Center Harwell; Paulsen lab (Uni. Of Cambridge)	RRID:MGI:6160916

**Software and algorithms**		

Arduino IDE 1.8	Arduino	https://www.arduino.cc/en/software
Python version 3.7.10	Python Software Foundation	https://www.python.org
DeepLabCut	Mathis et al.^23^	https://github.com/DeepLabCut
Scikit-learn	Pedregosa et al.^38^	https://scikit-learn.org/
Custom analysis code	This paper	Zenodo: https://doi.org/10.5281/zenodo.8003569

**Other**		

Arduino Mega 2560 Rev3	Arduino	https://www.arduino.cc/
Raspberry Pi 3 Model B	Raspberry Pi Foundation	https://www.raspberrypi.com/
2-Way NC Pinch Valve 12VDC	NResearch, Inc.	Part# 161P011
Stepper motor – Nema 17	Adafruit	Product ID: 324

### Resource availability

#### Lead contact

Further information and request for resources should be directed to and will be fulfilled by the lead contact, Julija Krupic (jk727@cam.ac.uk).

#### Materials availability

This study did not generate new unique reagents.

### Experimental model and study participant details

#### Mice

Experimental procedures and animal use were performed in accordance with UK Home Office regulations of the UK Animals (Scientific Procedures) Act 1986, following ethical review by the University of Cambridge Animal Welfare and Ethical Review Body (AWERB). All animal procedures were authorized under Personal and Project licences held by the authors.

Four groups of mice were used in the study: C57BL/6J mice with lesions to 1) the hippocampus, 2) the medial entorhinal cortex or 3) sham controls, with saline injections in the hippocampus, medial entorhinal cortex or medial prefrontal cortex. The fourth group comprised *App*^*NL-G-F*^^17^^,^^27^ mice (Table S1). All mice used in this study were males.

Eighteen C57BL/6J mice sourced from Charles River underwent lesion procedures. The experiments were carried out in two batches ∼3 months apart. The mice were 10–16 weeks old when they were transferred to the smart-Kages. They were individually housed in the smart-Kages for ∼30 days prior- and ∼60 days post-surgery. The mice weighed 25-30 g at the time of the surgery. Water and food were supplied *ad libitum*. The first batch was tested on the standard forced-choice alternation T-maze task, object recognition and object-in-place recognition tasks before and after testing in the smart-Kages. The second batch underwent the same standard tests only after their testing in the smart-Kages was completed. The mice were individually housed in clear plastic cages (16 cm × 27 cm × 18 cm, W × L × H) when they were tested on standard tasks (Figure S1E). They were maintained on a 90% body weight food restriction schedule when tested on the standard forced-choice T-maze alternation task. We also tested ten additional unlesioned 16-week-old C57BL/6J male mice for 30 days in the smart-Kages to test the generality of our clustering framework.

Three *App*^*NL-G-F*^ KI mice^17^^,^^27^ and three age-matched *App*^*wt/wt*^ KI negative controls were included in blinded test experiments. They were 22–24 weeks old when first tested in the smart-Kages. We also included two additional *App*^*NL-G-F*^ KI positive males aged 39 weeks whose identity was known. The mice were continuously tested for ∼4.5 months. The second testing period commenced ∼8 months later and lasted for ∼1.5 months. All eight mice were tested on the standard tests before and after they were tested in the smart-Kages.

All mice were kept on a 12:12 h light: dark cycle (with lights on at 9:00 a.m. and off at 9:00 p.m.) at a controlled temperature (21–23°C) and humidity (50–60%).

### Method details

#### Surgery

Mice were anesthetized with 1–3% isoflurane in O_2_, and 0.05mg/10g body weight Metacam and 0.05mg/10g Baytril was administered to facilitate recovery. Chemical lesions were induced by injection of 10μg/μL ibotenic acid dissolved in pH7.4 PBS into selected brain regions using a Nanofil syringe controlled by the micropump. To induce hippocampal lesions, we used the same coordinates and injected volumes as previously described in Voikar et al.^9^ To induce mEC lesions, we aimed to inject the following four coordinates bilaterally. mEC1 (150 nL): AP: 0.4 mm anterior to sinus; ML: 3.4 mm from the midline; DV:2.4 mm; mEC2 (150 nL): AP: 0.4 mm anterior to sinus; ML: 3.4 mm from the midline; DV:1.6 mm; mEC3 (150 nL): AP: 0.4 mm anterior to sinus; ML: 2.8 mm from the midline; DV:3.0 mm; mEC4 (150 nL): AP: 0.4 mm anterior to sinus; ML: 2.8 mm from the midline; DV:2.2 mm. The injection syringe was tilted at 6° anterior-to-posterior angle. Following surgery, mice were individually housed in a conventional cage, and their health conditions were monitored for six days before they returned to their corresponding smart-Kage when fully recovered.

#### Blinding procedures

The brain regions targeted for lesioning were known only to the researcher who conducted the lesion surgeries. Mice were selected at random, and their identities were unknown. Experimenters responsible for conducting behavioral tests, maintenance of smart-Kages, data collection and analysis were blinded to the lesion identity and groups for the duration of the experiments. The genotypes of three homozygotes *App*^*NL-G-F*^ mice and three control littermates were kept hidden from all the experimenters until the analysis was completed. The identities of two older homozygote *App*^*NL-G-F*^ mice were known to the researchers.

During the lesion quantification from the histology, the experimenter was blind to the animals’ characterization in the smart-Kage or their performances on the standard tests.

#### Histology

Following completion of the experiments, the mice were given an overdose of sodium pentobarbital and perfused transcardially with phosphate-buffer saline (PBS), followed by 4% formaldehyde to fixate the brain tissue. The brains were carefully extracted from the skull and stored in 4% paraformaldehyde (PFA) at 4°C. Brains were then imaged using serial two-photon tomography, which sliced and imaged the entire brain every 20 μm coronally with a resolution of 4 μm in x and y using autofluorescence at 800 nm. To estimate mEC lesions, brains were resliced computationally into sagittal sections. Lesion volume was estimated by manually marking the total brain area volume and the lesion volume every 60 μm (for HP lesions) and 50 μm (for mEC lesions).

#### Smart-Kage design

The smart-Kages and associated components were designed using computer-aided design software. The smart-Kages were manually assembled from parts made of 3- and 5-mm thick transparent acrylic sheets that were laser-cut into correct dimensions and designs. The final dimensions of smart-Kage were 39 cm × 32 cm x 44 cm (W x L x H). The smart-Kage was fastened to a base and flanked by two drums used for NOR and OPR tasks (see above). An overhead infra-red camera was installed on a removable lid, providing a top view into the Kage interior and was used for continuous video recording at two frames/second. Infra-red LEDs were distributed around the lid to provide illumination. The smart-Kage was fitted with two pairs of beam breakers positioned near the water spouts to detect when a mouse attempted to engage in drinking behavior and trigger a solenoid valve if the approached spout was ‘correct’ (i.e., different from the previously visited water spout). The two solenoids were placed on the outer sides of the side walls and connected to nearby attached small water containers via a thin tube. The beam breakers and solenoids were connected to and controlled by a single-board microcontroller attached to the outer part of the back wall of the smart-Kage. The microcontroller was also connected to and controlled the rotation of the drums used for NOR and OPR tasks. The drums were only rotated when the mouse was in one of the corridors engaged in ‘drinking behavior’ (or displaying an attempt to drink) so that a mouse could not directly observe the rotation of the drums. The rotation timing was programmed by the experimenter – although the exact timing depended on when a mouse engaged with any of the water spouts.

All data generated was automatically transferred to a single-board computer for data sorting and storage (see below). The smart-Kage contained a running wheel, a climbing platform and nesting material. The smart-Kage included three integrated cognitive tasks: the smart spontaneous T-maze task, smart NOR and OPR tasks. Each mouse interacted with the tasks of its own volition, and data was continuously gathered using sensors and a video camera.

#### Experimental procedures

Mice were group-housed and acclimatized in the holding facility for at least one week prior to the start of experiments. Before transferral to the smart-Kages, the first batch of C57BL/6J and *App*^*NL-G-F*^ mice underwent a series of standard behavioral tasks, comprising forced-choice alternation T-maze task, novel object recognition (NOR) and object-in-place recognition (OPR) tests. All mice participated in the same set of tests following the smart-Kage experiment. The standard testing protocols were based on published versions in the literature^11^^,^^14^ and briefly described below.

#### Testing in the smart-Kages

Mice were kept single-housed in the smart-Kages with free access to food and water. During the habituation stage (5–7 days), mice received water from both spouts. After the habituation period, a smart spontaneous alternation T-maze task commenced when only one of the water spouts (an active spout) provided water at any given time. The location of the active water spout alternated every time a mouse received the water (i.e., accessed an active spout). The smart NOR and OPR tests were implemented by rotating the side drums to present one of the eight side patterns (0.5 cm × 6.4 cm x 9.2 cm) accessible for a mouse to explore. The drum rotation was programmed to occur every two days between 12 a.m.–4 p.m. and occurred only when a mouse was drinking water in one of the corridors to ensure that it could not observe the change. The patterns were presented according to a schedule designed to test the mouse’s spatial and non-spatial ‘object’ recognition abilities. The drum-change sequence consisted of the following combinations: (1) left-drum only NOR, (2) right-drum only NOR, (3) both drums NOR, (4) left-drum only OPR, (5) right-drum only OPR, (6) both drums OPR, (7) left-drum NOR/right-drum OPR, and (8) right-drum NOR/left-drum OPR.

Mice underwent lesion surgery after 4–6 weeks of residing in the smart-Kages and were kept in conventional cages for six days of post-operative care. Following full recovery from surgical procedures, mice were transferred back to the same smart-Kages and kept for 8–12 weeks. The smart-Kages were cleaned every two weeks. Mice were tested in standard behavioral tests following the smart-Kage experiment, as described below.

#### Standard forced-choice alternation T-maze task

The test was conducted using a T-shaped enclosure consisting of a start arm adjoining two perpendicular goal arms. Mice were food-restricted for at least 12 h before each experiment day and were kept at approximately 90% of initial body weight. Soya milk was used as a reward and was located in a food well at the end of the goal arms. One day prior to the testing session, mice were habituated to the apparatus by allowing them to freely explore the enclosure. The habituation consisted of four 3-min periods of exploration, interleaved by a 10-min interval. During the habituation, mice could drink soya milk *ad libitum* from the food well at both goal arms.

Each daily session was composed of 10 trials, and each trial consisted of a sample run followed by a test run. Each pair of the sample and test runs were separated by about 12–15 min. During the test run, the mouse was kept in the start arm for 10 s before exploring the goal arms. If the goal arms were not visited within 90 s, the mouse was removed, and the trial was terminated.

#### Standard NOR and OPR tests

We closely followed the protocol described by Leger et al. (2013).^14^ Namely, the tests were performed in a 0.5 × 0.5m^2^ square enclosure. Objects were placed 15 cm from the walls. Object exploration was defined as instances when the mouse looked or sniffed at the object in proximity (<2 cm) or when there was direct contact with a snout or paws. Climbing or chewing was not counted as object exploration. One day before the tests began, the mice were allowed to freely explore the enclosure without any objects present. The habituation consisted of two 10-min periods of exploration, with a 3-h interval in between each period. The tests comprised a familiarisation session followed by a test session with a 2-h inter-trial delay. For NOR, two objects were included in each session, and for OPR, two pairs of objects were used. The familiarisation session was run for 5 min unless a mouse explored an object for over 40 s, at which point the mouse was removed and the trial terminated. In NOR, one of the two objects was replaced with a new one between sessions. The other object was replaced with an identical object to ensure familiarity was not based on a mouse marking the familiar object. In the OPR task, all objects were replaced with identical objects during the test trial, and the positions of one pair were swapped between sessions. An overhead camera was used to capture videos of mouse activity for post-hoc visual inspection and analysis.

#### Smart-Kage data collection

Parameters and functions of smart-Kage sensors and motors were configured using a custom-written script. An overhead IR camera was used to capture videos of mouse activity at two frames per second (temporal resolution of 0.5 s). Additionally, every approach toward the water spouts (both correct and incorrect choices) was relayed by beam-break sensors to the controller. Time was synced directly from the internet. All data generated was instantaneously transmitted to a computer for storage, logging and sorting.

#### Mouse video tracking

The mouse location was tracked from recorded videos using a ResNet-101 deep convolutional neural network (CNN). The starting architecture (pre-trained on ImageNet) was retrained for mouse tracking within the smart-Kage using transfer learning implemented in DeepLabCut (DLC)^23^ software and a dataset of video frames manually labeled with eight mouse body parts (snout, left and right ears, neck, 3 points along the mouse’s spine and tail base; Figure 1B).⁠ The retraining was done in 9 consecutive cycles with the ADAM optimizer, batch processing (batch size 8) and *imgaug* image augmentor.^23^^,^^39^ In each consecutive training cycle, the dataset was manually expanded with frames on which the resulting network from the previous cycle performed poorly. The expanded dataset was then randomly split into train and test subsets (95% and 5%, respectively), the network trained on the training subset and its performance evaluated on the test subset. The random splitting of the dataset was repeated three times within each cycle (generating three different “shuffles” of train and test datasets, Figures S2A and S2B) to safeguard against overestimated network performance due to a favourably chosen test subset. The network performance in each cycle was estimated as the average test dataset error (MAE between predicted and ground truth mouse body-part labels) across all 3 test shuffles. The cycles were repeated until network performance plateaued in cycle nine at 1.69 px (spatial resolution of 1.85 mm; Figure S2C). A total of 800 manually labeled frames were used.

#### Behavioral labeling

Behavioral labels (e.g., ‘exploring NOR’, ‘T-maze trial’, ‘quiescence’ etc.) were assigned to mouse trajectories and body postures in three main ways. Smart T-maze trials were assigned whenever a mouse presence in the corridors coincided with beam-breaker detection. Sleeping was assigned to frames with little or no detected motion, cross-validated with frame subtraction. Finally, pattern exploration and running-wheel exercise were assigned with a random forest classifier. The classifier hyperparameters were tuned with randomized 3-fold cross-validation, and the classifier was subsequently trained on a training dataset of video frames manually labeled with ground-truth behavioral labels. Specifically, a 32-dimensional feature vector was extracted from each frame, containing (x, y) coordinates and (|v_x_|, |v_y_|) absolute speeds for all eight body parts. The body-part speeds were calculated as position differentials between two subsequent frames (v_x_ = x_t+1_-x_t_, v_y_ = y_t+1_-y_t_), followed by median averaging with a rolling window of 81 frames (40 s; Figure S2D). The chosen features explained 95% of the variance in the training dataset (Figure S2E). The classifier performance for each behavioral category was estimated on the test dataset, as shown in Figure 1D. A total of 542,530 manually labeled frames were used, with an 80%: 20% random train: test dataset split.

#### Feature PCA analysis

We tested which behavioral measurements used for clustering analysis were the most important in differentiating between animal phenotypes (explaining the variance in the dataset) with Principal Component Analysis (PCA; Figures S2F–S2I). The first two principal components by definition explain the largest percentage of variance in a given dataset; in our case, the first two PCA components explained 33% of total variance (Figure S2G). T-maze and quiescence behavioral features were the most relevant in the first PCA component (Figure S2I, top), whereas the second PCA component was explained largely with NOR/OPR features (Figure S2I, bottom), indicating that no single behavior was sufficient to distinguish between animal phenotypes.

#### Feature correlation analysis

We tested whether behavioral measurements used for clustering analysis were correlated by computing their pairwise Pearson’s r correlation coefficients. The correlation coefficients were computed for all pairs of 32 behavioral measures across all mice. This resulted in a 32x32 correlation matrix, with Pearson’s r coefficients ranging from −1 (strong negative correlation) to +1 (strong positive correlation) (Figure S10A). Significantly correlated pairs of behavioral measurements were identified as the ones whose absolute Pearson’s correlation coefficient value was above the threshold, determined as the 95^th^ percentile of correlation values, obtained from randomized data. Data was randomized by randomly shuffling behavioral measurements across mice without mixing different types of measures (Figure S10B). The shuffling process was repeated 10,000 times, and the 32x32 matrix of 95^th^ percentile threshold correlation values was calculated (Figure S10C). Finally, we identified significantly correlated measures by subtracting the 32x32 threshold matrix from the corresponding absolute values of the behavioral correlation matrix. All values above zero indicated a significant correlation between pairs of behavioral measures (Figure S10D).

#### Mouse group assignment in smart-Kages

We aimed to minimize human bias by optimizing clustering parameters within the blinded experimental framework. To achieve this, we took advantage of one ‘known’ group within the otherwise blinded experimental dataset. Namely, all pre-lesioned mice represented a known control group because prior to the lesion procedures (for which we were blinded), all three groups (HP, mEC and sham control) consisted of C57BL/6J mice of similar age and same gender and were broadly expected to show similar ‘normal’ behavioral phenotypes. Therefore, in our case, a part of the dataset (i.e., pre-lesioned control mice) is labeled, and we are dealing with a semi-supervised problem. To include this information in the training of our clustering algorithm, we ran 25,000 clustering simulations with our data (32 behavioral features for all mice, not just controls). In every simulation, we tested a different combination of a clustering algorithm and its associated hyperparameters. The following common clustering algorithms were tested: K-means, Bayesian Gaussian mixture models, agglomerative clustering, OPTICS, spectral clustering and affinity propagation. Next, we chose a subset of clusterings that identified the only *a priori* known group (the ‘control’ mice) with 100% accuracy. The final clustering was chosen from this subset after unblinding the remaining group identities (HP, mEC, sham controls and *App*^*NL-G-F*^). We found that agglomerative clustering using ward linkage and Euclidean distance metric showed the highest accuracy in identifying these groups. The accuracy was measured as the percentage of correctly identified mice (Figure 7C). It should be noted that no additional clustering simulations were run after unblinding; i.e., the optimal clustering was chosen from among simulations done before unblinding to minimize human bias.

Next, we tested the clusters’ quality and stability by applying a leave-one-animal-out approach. We ran 10,000 clustering simulations, in which one randomly-chosen animal was removed from the dataset and the same clustering repeated on the remaining dataset. The resultant clusterings were compared with the original clustering using the mean Silhouette score^32^^,^^33^^,^^38^ and the chance-adjusted Rand Index (RI) score^34^^,^^35^ to quantify the quality and stability, respectively. The mean Silhouette score ranges from −1 (worst) to +1 (best), where positive values indicate good separation (a high quality) between clusters, and negative values indicate that a mouse was assigned to the wrong cluster and is hence an outlier. Values close to zero signal overlap between clusters. The estimated mean Silhouette score of our clustering was equal to 0.1547 ± 0.0928 (mean ± std). The RI score ranges from 0 (random labeling) to 1 (identical clusters). The estimated mean RI value of our dataset was 0.8926 ± 0.1463 (mean ± std).

To test the ‘goodness’ of such clustering quality and stability, we compared it with the Silhouette score and RI obtained from the randomized data (Figures S2J and S2K). The randomized data was generated by randomly shuffling existing features across mice within each feature type (e.g., T-maze feature cannot be swapped with the locomotion feature, Figure S10B). Good clustering stability was defined as the one with the Silhouette and RI scores higher than a chance threshold value calculated as the 95^th^ percentile value of the surrogate data. Each surrogate dataset generation was repeated 10,000 times. The resultant chance Silhouette score threshold was 0.1137 (mean ± std: 0.0564 ± 0.0293), while the resultant chance RI score was equal to 0.1243 (mean ± std: 0.001 ± 0.0666). These values indicate a good deviation of our original clustering from randomness.

#### Mouse group assignment based on standard memory tests

We used the performance measurements from standard T-maze, NOR, and OPR tasks to group the mice on an individual animal basis to benchmark the prediction of the smart-Kage against analogous standard tests (Figure S4F). 42.86% (12/28) and 46.43% (13/28) of mice were discarded in NOR and OPR tests, respectively, at a 30% threshold difference between the exploration of both objects during the familiarisation session. The remaining mice were clustered with simple threshold criteria. Similar to the smart-Kage clustering above, these thresholds were selected to assign a maximum number of control mice into a single ‘control’ cluster. Mice with T-maze performance below 70%, the absolute value of NOR d2 ratio below 0.04 and the absolute value of OPR d2 ratio below 0.06 were identified as displaying cognitive decline. In brief, the d2 ratio is the difference in exploration time between the novel and familiar object, normalized with respect to their combined exploration time. Hence, a d2 value of 0 indicates equal exploration time between the two objects, whereas values closer to +/−1 indicate a preference for one of the objects.

### Quantification and statistical analysis

The effects of different lesions were tested by statistically comparing pre- and post-lesion periods of equal time spans (∼30 days) within each group (control, HP and mEC mice) independently; in the case of translational *App*^*NL-G-F*^ mice, periods of different mice age (i.e., 5–9 months vs. 18–20 months) were compared instead.

First, the normality of our data was checked using the Shapiro-Wilk test.^40^ Since the majority of our measures were found to be normally distributed, we proceeded with parametric statistical tests unless otherwise stated. Specifically, the mean value of a given behavioral measure (averaged across days) was calculated for each mouse, and all combined pre-lesion means were compared to their post-lesion counterparts with paired samples Student’s t-test. In the case of non-normally distributed data, the non-parametric Wilcoxon signed-rank test was applied. A one-way ANOVA with repeated measures correction was used when comparing animal reaction times to changes in drum patterns between the three lesion groups. All p-values were corrected for multiple comparisons within each behavioral type (Table S2) using the Benjamini-Hochberg correction. All data presented is reported as mean ± s.e.m. unless stated otherwise.

Python was used for all statistical calculations^41^; paired samples Student’s t-test: *scipy*.*stats*.*ttest_rel*^42^; Wilcoxon signed-rank test: *scipy*.*stats*.*wilcoxon*; one-way ANOVA: *statsmodels*.*stats*.*anova*.*AnovaRM*.^43^

## Data Availability

• Processed data have been deposited at Zenodo and are publicly available as of the date of publication. DOIs are listed in the key resources table. The training data for NN models for tracking cannot be made publicly available since it is a proprietary dataset. The raw image data required to reanalyse the processed data is available from the lead contact upon reasonable request. • All original code has been deposited at Zenodo and is publicly available as of the date of publication. DOIs are listed in the key resources table. • Any additional information required to reanalyse the data reported in this paper is available from the lead contact upon reasonable request.

## Supplemental information

Document S1. Figures S1–S10 and Tables S1 and S2

Document S2. Article plus supplemental information
