# Supplementary material for: A fully automated home cage for long-term continuous phenotyping of mouse cognition and behavior
Source: Cell Rep Methods. 2023 Jul 13;3(7):100532. doi: 10.1016/j.crmeth.2023.100532 (PMC10391580; doi:10.1016/j.crmeth.2023.100532)
Supplement: Document S1. Figures S1–S10 and Tables S1 and S2 [file mmc1.pdf]

Cell Reports Methods, Volume *3*

Supplemental information

A fully automated home cage for long-term continuous phenotyping of mouse cognition and behavior

Hinze Ho, Nejc Kejzar, Hiroki Sasaguri, Takashi Saito, Takaomi C. Saido, Bart De Strooper, Marius Bauza, and Julija Krupic

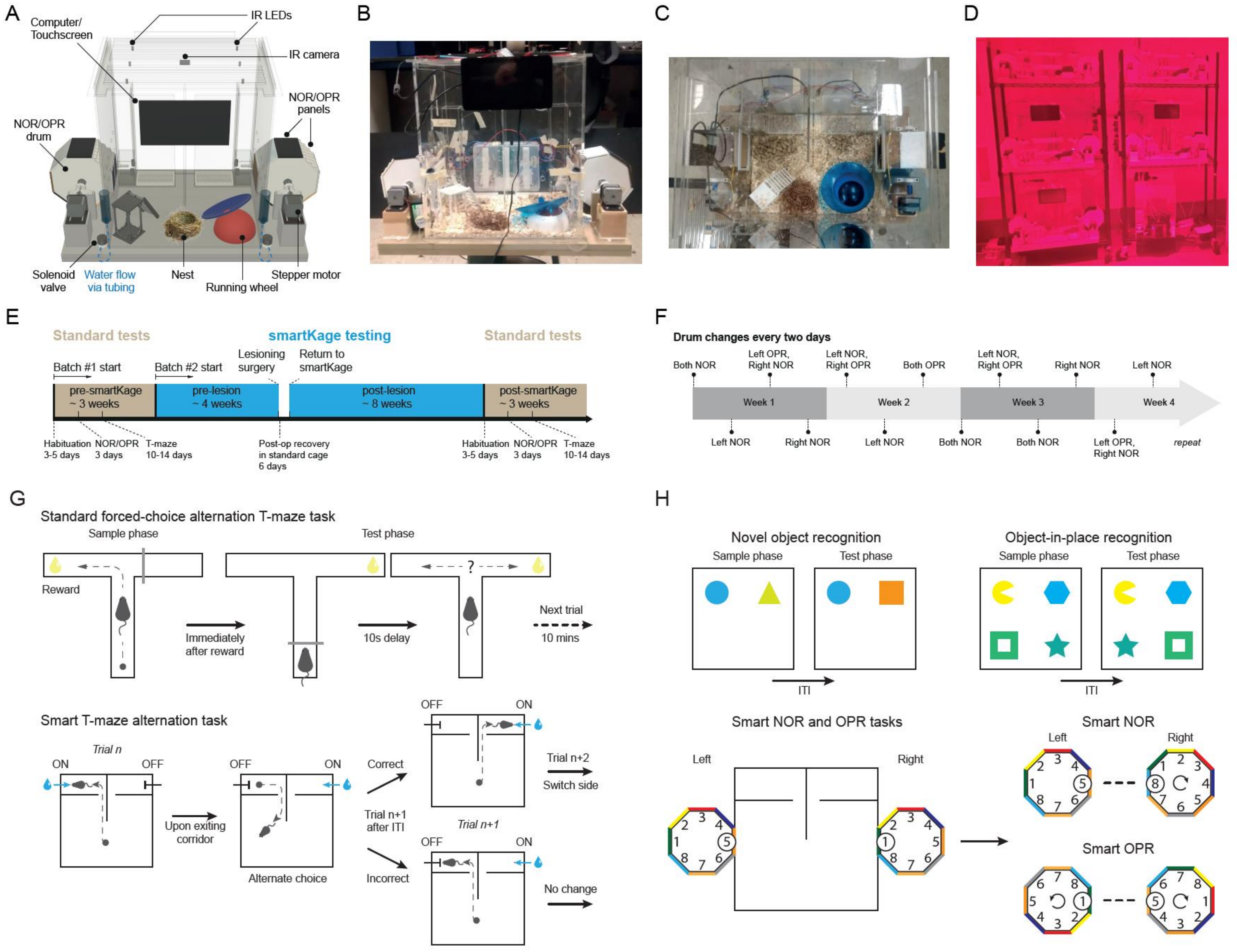

Figure S1. The smart-Kage design and schematic representation of standard memory tasks and their analogous tasks in the smart-Kage, related to Figures 1-3. **(A)** A schematic of the smart- Kage home monitoring system. The schematic is up to scale to facilitate replication (see “Smart-Kage design” section for exact dimensions). **(B)** A picture of the smart-Kage home monitoring system, as shown in the schematic (A). **(C)** A top-view picture of the smart-Kage. **(D)** Six smart-Kages are shown during the experiment. **(E)** Experimental timeline for batch #1 & #2 smart-Kage experiment. **(F)** A basic drum sequence. For longer testing periods, the sequence was repeated. **(G)** Top: a standard forced-choice T-maze alternation task explained. A mouse is run in a T-shaped enclosure with one of the arms blocked and a few drops of soya milk (a ‘reward’) placed at the end of another arm. After consuming the reward, a mouse is placed at the start of a T- maze with the arm closed for a fixed duration. On its release, the mouse has to alternate the arm to receive the reward. In the smart T-maze alternation task (bottom), the water is available only on one of the waterspouts at the end of the left or right corridor. After a mouse consumes the water, it must choose the opposite side to receive it next time. The action is voluntary, and hence ITIs vary from several seconds to hours. **(H)** Top: in the standard NOR task, a mouse is presented with two novel objects (shown as a circle and a triangle) during a ‘sample phase’. After a fixed ITI, a mouse is placed back and is presented with one previously seen and one new object. Normally, mice spend more time at the novel object. Standard OPR task is similar to standard NOR object. However, here two pairs of objects are presented. One pair of objects have the locations of two objects swapped. A normal mouse will spend more time at objects whose locations were swapped. In the smart NOR task (bottom), one of the drum patterns is changed to a new pattern. A normal mouse spends more time at a novel pattern. Both patterns remain the same in the smart OPR task, but their locations are swapped. Different patterns are represented with different colors.

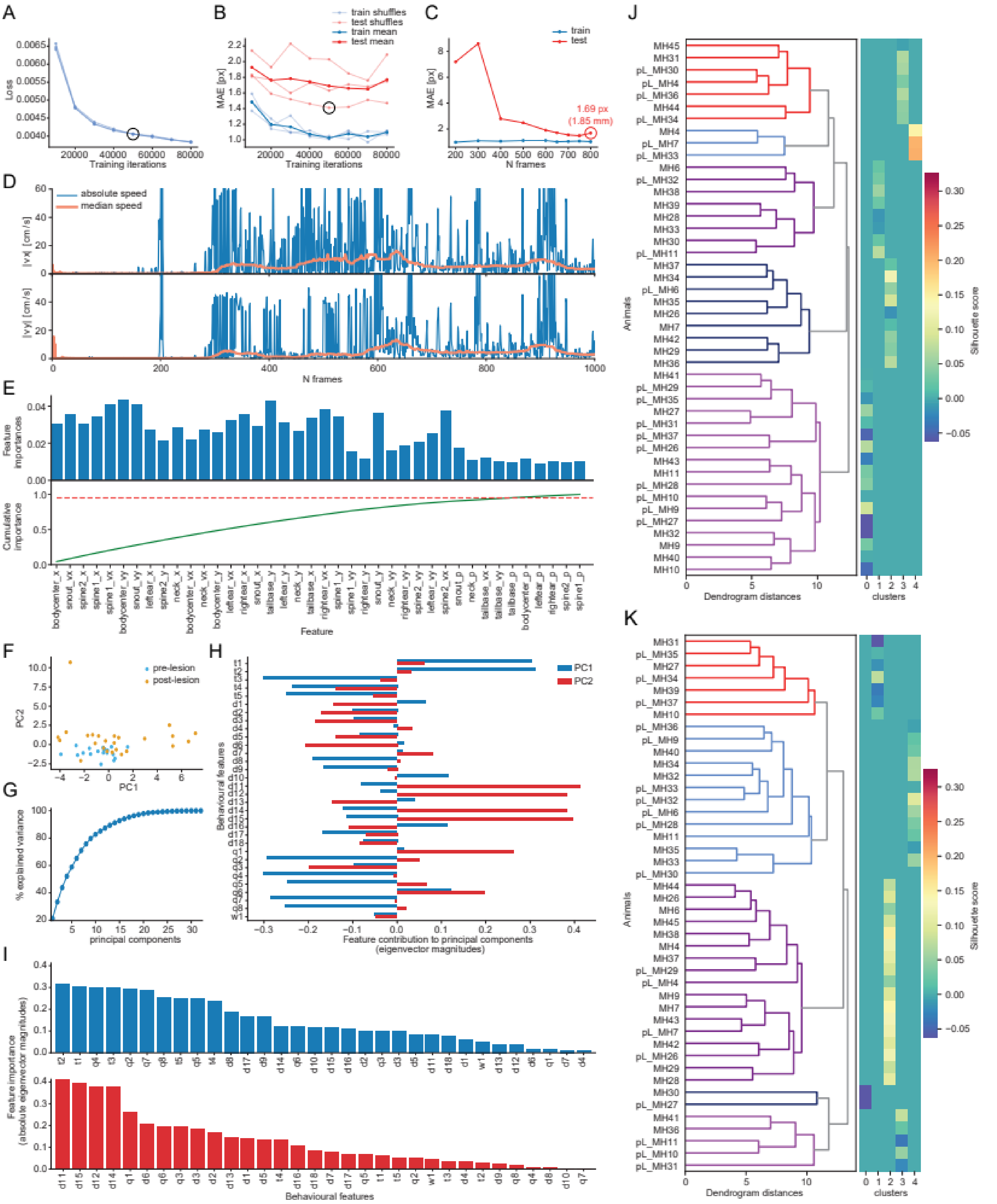

A
Loss
Training iterations
B
train shuffles
test shuffles
train mean
test mean
MAE [px]
Training iterations
C
train
test
1.69 px
(1.85 mm)
MAE [px]
N frames
D
absolute speed
median speed
|vx| [cm/s]
|vy| [cm/s]
N frames
E
Feature importances
Cumulative importance
Feature
F
pre-lesion
post-lesion
PC2
PC1
G
% explained variance
principal components
H
PC1
PC2
Behavioural features
Feature contribution to principal components
(eigenvector magnitudes)
I
Feature importance
(absolute eigenvector magnitudes)
Behavioural features
J
Animals
Dendrogram distances
clusters
Silhouette score
K
Animals
Dendrogram distances
clusters
Silhouette score

Figure S2. Details of smart-Kage software implementation, related to Figures 1 and 7. (A-C) CNN training with DeepLabCut (DLC). **(A)** An example learning- loss plot from a single training cycle with 80,000 training iterations. The black circle indicates a plateau in learning loss, at which point the network weights for the next training cycle were taken. **(B)** An example plot from a single training cycle with 80,000 training iterations showing the network performance evaluation on the train (blue) and test (red) subsets (95% and 5% of the original training dataset, respectively). The random train-test splitting of the dataset was performed three times during each cycle, generating shuffles of train and test subsets (light blue and red, respectively). The performance is measured as the mean absolute Euclidean distance (MAE) between human-annotated and network-predicted body part labels in units of pixels, averaged across all shuffles (dark blue and red, respectively). Unlike in root mean square error (RMSE), in MAE the Euclidean distance is calculated before calculating the mean over frames and body parts (i.e. operations of square root and mean are swapped). The black circle indicates a minimum in the test subset MAE of one shuffle, at which point the network weights for the next training cycle were taken. **(C)** Neural network training progression across all training cycles. The network was trained until the test dataset MAE plateaued at 1.69 px (9 cycles, 800 total training frames). **(D)** Median speed averaging. The blue line demonstrates absolute speed calculated as the difference in position between two subsequent frames. The orange line shows the median average, calculated in an 81-frame (40-second) window over the blue line. The median value was assigned to the central frame of the rolling window (frame 41). The top and bottom plots show x- and y-axis speeds, respectively. **(E)** Importances of individual features (top) and cumulative importance of all features (bottom) used in RF classification. “bodypart_x”, “bodypart_y” - x,y coordinates of “bodypart”. “bodypart_vx”, “bodypart_vy” - absolute speeds of “bodypart” along x- and y-axis, respectively. “bodypart_p” - DeepLabCut (DLC) prediction certainty for (“bodypart_x”, “bodypart_y”) prediction. DLC prediction probabilities were not used as part of the feature vectors, as 95% of explained variance (red dashed line) was explained with coordinate and speed features. **(F-I)** PCA analysis of the behavioural feature space. **(F)** First two dimensions (principal components) of PCA-transformed behavioural feature space. Each data point represents a 2-dimensional embedding of a 32-dimensional behavioural feature vector obtained from a single smart Kage. Blue and orange points originate from pre- and post-lesion periods, respectively. **(G)** Percent of cumulative dataset variance, explained by individual principal components. **(H)** Signed feature contributions (eigenvector magnitudes) to the first two principal components (blue and red, respectively). **(I)** Absolute feature contributions (absolute eigenvector magnitudes) to the first (top, blue) and second (bottom, red) principal components, ordered from largest to smallest. tX - tmaze features, dX - NOR/OPR features, qX - quiescence features, wX - running-wheel features (see Table S2 for feature details). **(J-K)** Clustering randomization. **(J)** An example of clustering with randomly generated features. Each of the 32 features for every animal was sampled from a normal distribution with 0 mean and unit variance, in line with standardized features used in the original clustering. **(K)** An example of clustering with randomly shuffled features. The features were shuffled only between animals and not across different feature types. The silhouette scores are displayed in the same range as in the original clustering in Figure 7 for comparison.

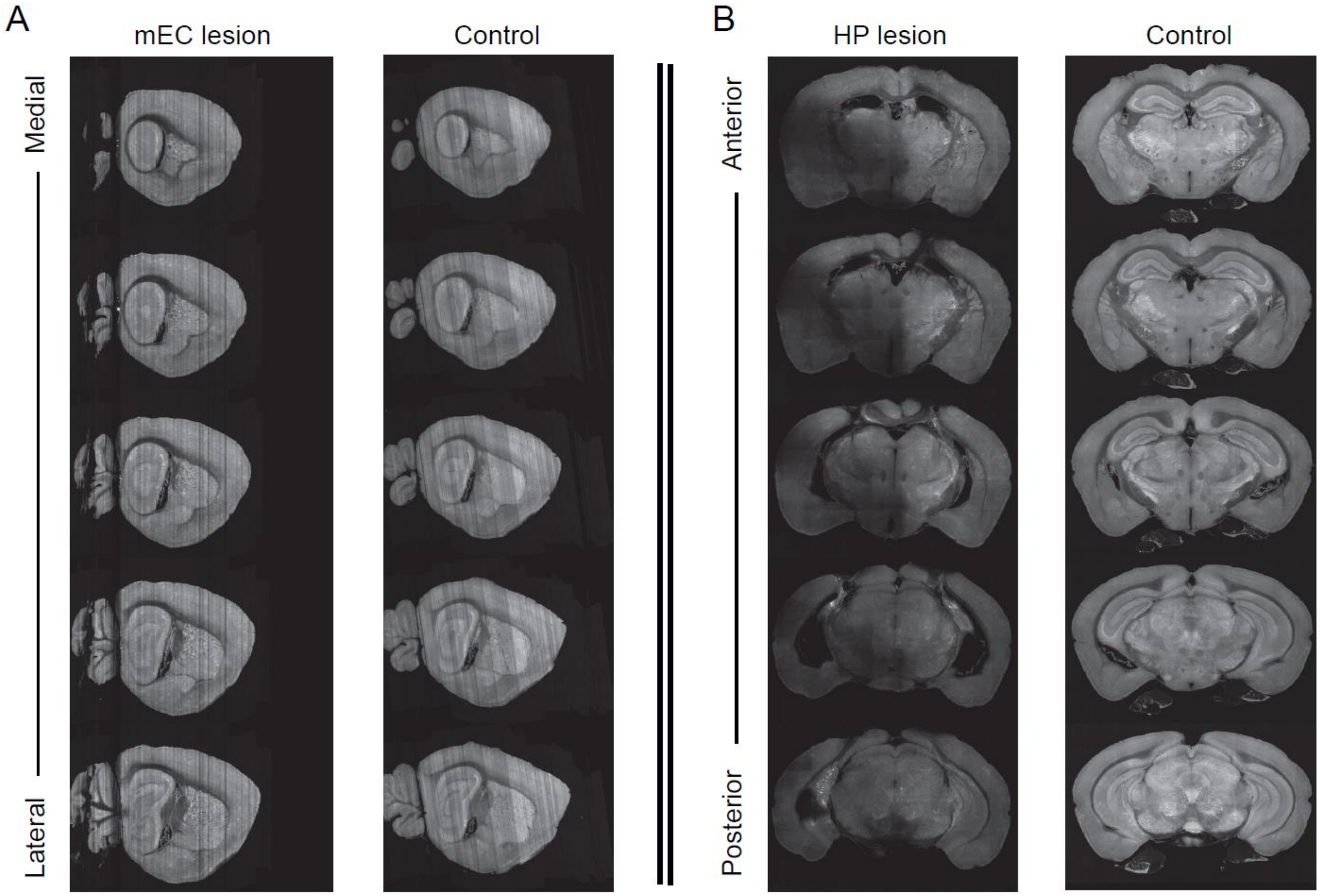

C

Lesion site	Mouse ID	Average combined lesion size (%)	Left hemisphere lesion size (%)	Right hemisphere lesion size (%)
HP	MH26	56	67	44
	MH27	52	58	46
	MH28	52	38	66
	MH29	67	82	52
	MH31	49	54	43
mEC	MH04	20	12	28
	MH09	65	92	38
	MH30	97	97	97
	MH33	52	60	44

Figure S3. Histology, related to Figures 2 and 7. (A) An example of mEC lesion. **(B)** An example of HP lesion. **(C)** Identities of hippocampal- and medial-entorhinal-lesioned animals and corresponding quantification of lesion size.

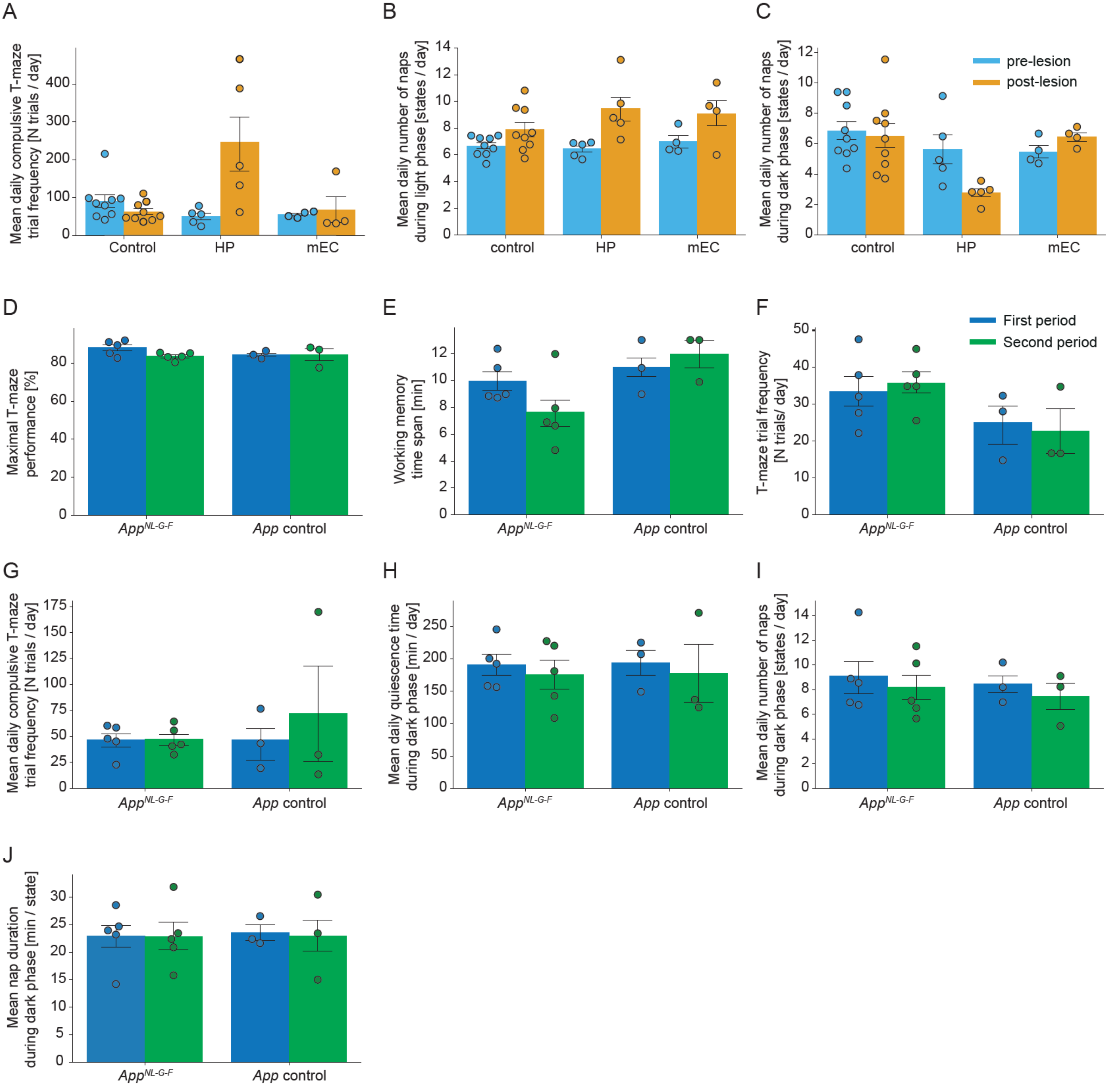

Figure S4. Statistical tests on additional behavioural features, which were found to be non-significant between test groups, related to Figures 2,5,6. (A) Mean ‘compulsive’ T-maze trial frequency of lesioned animals. ‘Compulsive’ T-maze trials are defined as the animal attempting to drink multiple times without leaving the T-maze corridor. **(B)** Number of quiescent states per day (quiescence frequency) during light phase of lesioned animals. **(C)** Number of quiescent states per day (quiescence frequency) during dark phase of lesioned animals. **(D)** Maximum T-maze performance of *App*$^{NL-G-F}$ animals. **(E)** Memory time-span of *App*$^{NL-G-F}$ animals. **(F)** Maximum number of daily drinking attempts of *App*$^{NL-G-F}$ animals. **(G)** Mean compulsive T-maze trial frequency of *App*$^{NL-G-F}$ animals. Compulsive T-maze trials are defined as the animal attempting to drink multiple times without leaving the T-maze corridor. **(H)** Total quiescence time during dark phase of *App*$^{NL-G-F}$ animals. **(I)** Number of quiescent states per day (quiescence frequency) during dark phase of *App*$^{NL-G-F}$ animals. **(J)** Average duration of quiescent states during dark phase of *App*$^{NL-G-F}$ animals.

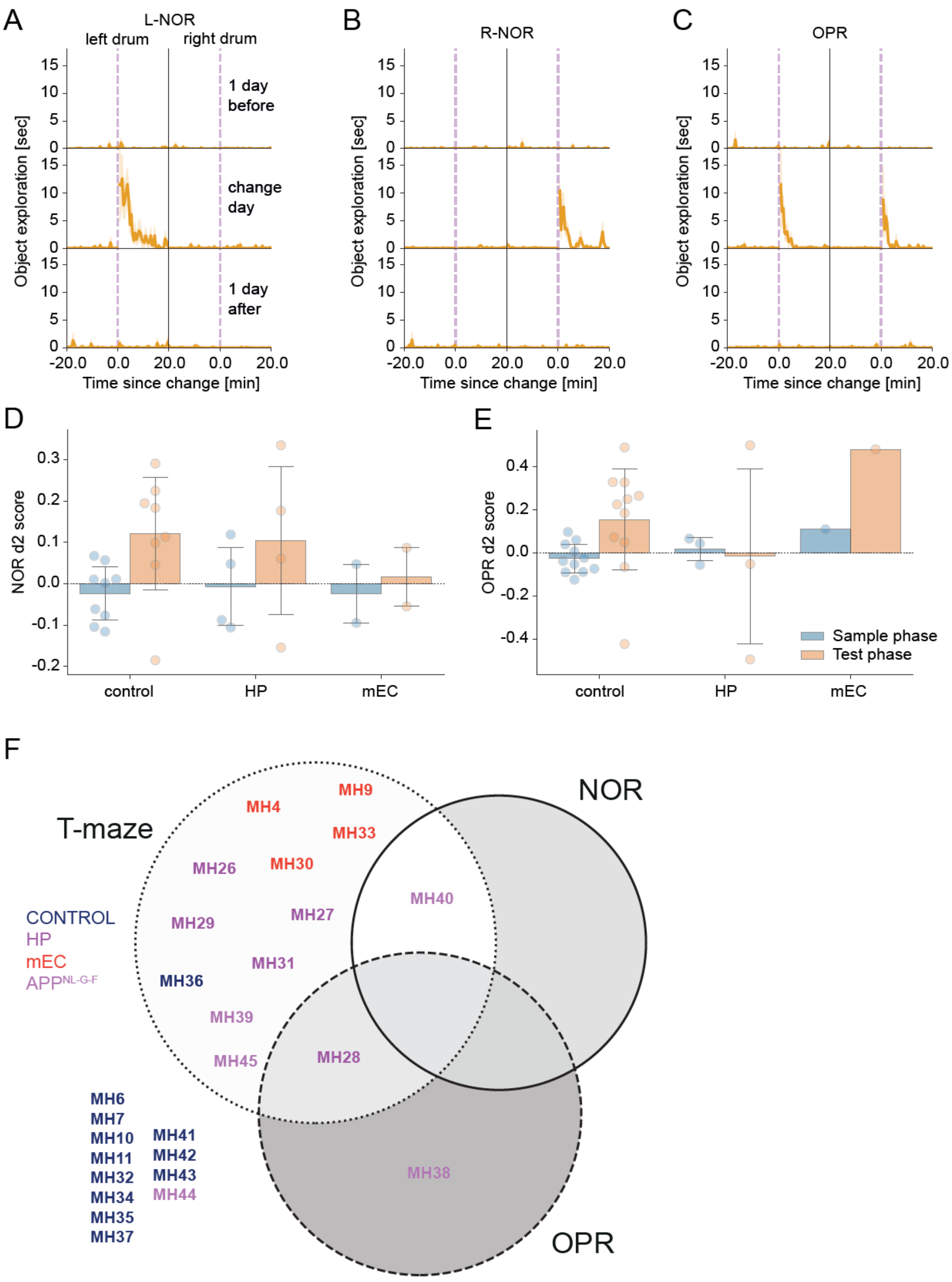

Figure S5. A representative example of NOR and OPR trials in the smart-Kage and results of the standard NOR and OPR tests, related to Figure 3. Left-NOR task **(A)**, Right-NOR task **(B)**, and OPR task **(C)** in pre- lesioned WT mice. Object exploration was measured at approximately the same time (~12 pm) one day before the change (top row), on the day of the change (middle row) and one day after the change (bottom row). A dashed pink line indicates the time of the change. **(D)** Standard NOR test. The differences between d2 scores during the test sessions are not significant between the groups (control vs. HP: t=0.1584, P=0.8773; control vs. mEC: t=0.9403, P=0.8773; HP vs. mEC: t==0.5523, P=0.8773; paired samples Student's t-test). **(E)** Standard OPR test. Similarly, the differences between d2 scores during the test sessions are not significant between the control and HP group (control vs HP: t=0.8651, P=0.4039; paired samples Student's t-test; statistical comparison with mEC group is not possible, as it contains a single sample). The normality of the data was verified with Shapiro-Wilk test and P-values adjusted for false discovery rate with Benjamini-Hochberg correction. Blue: familiarization session; orange: test session; dots - individual mice. Data are represented as mean ± SEM. **(F)** Mouse group identification on an individual animal basis using standard T-maze, novel-object recognition and object-in-place recognition tests. Different colours represent different mice groups. The Venn diagram contains mice that did not pass the high-performance thresholds in respective standard tests. The thresholds were selected to optimize the number of mice correctly identified as the control group shown outside the Venn diagram.

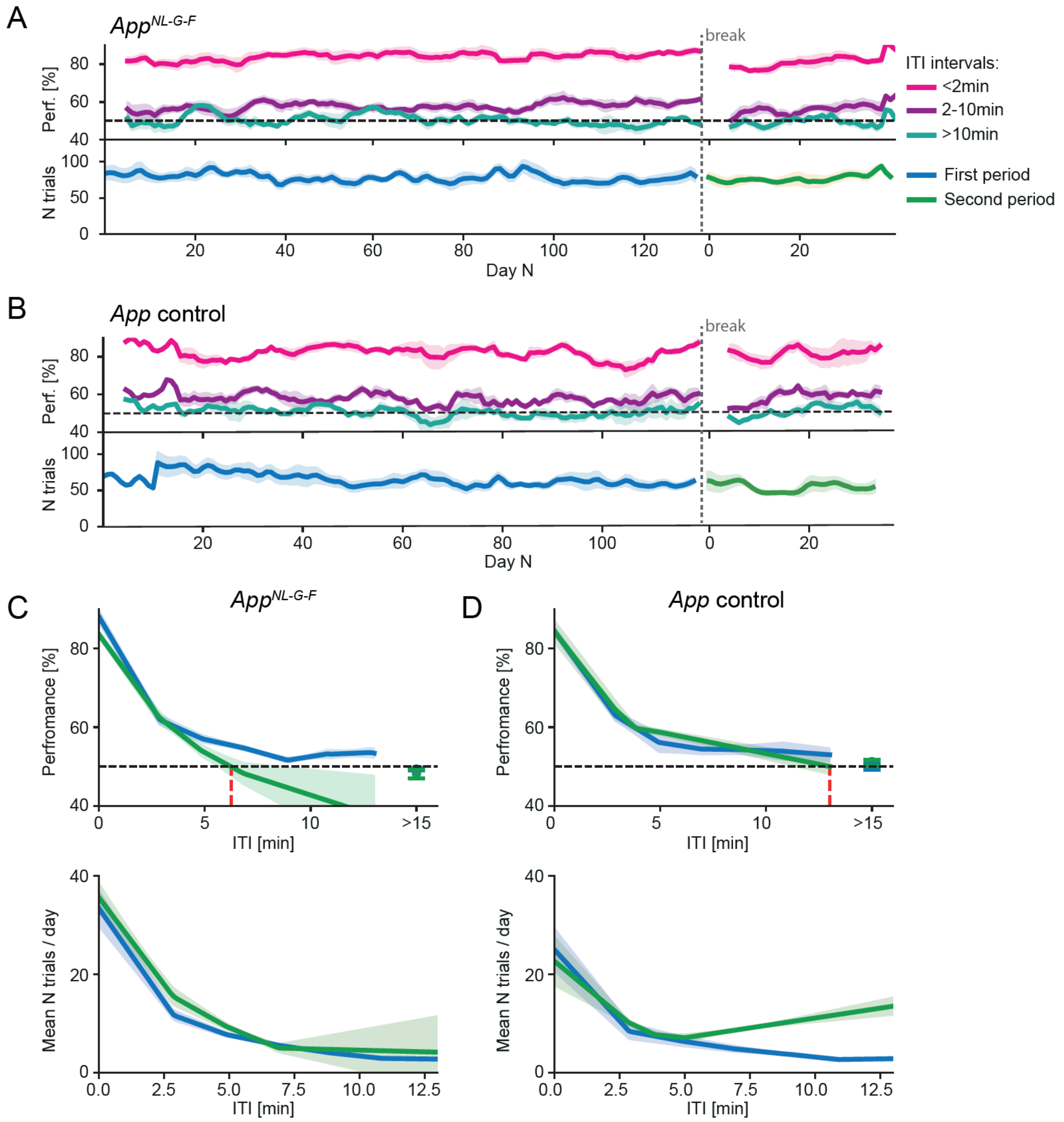

Figure S6. Smart T-maze spatial alternation task on *App$^{NL\text{-}G\text{-}F}$* animals, related to Figure 6. (A,B) Running average performance at different ITI intervals (top) and frequency of spout visits (bottom) in *App$^{NL\text{-}G\text{-}F}$* (A) and age-matched control (B) mice at 5-9 months and 18-20 months of age, separated by the vertical dashed line (labelled "break"). The horizontal dashed line indicates chance level performance. **(C,D)** The average distribution of performance (top) and daily frequency of spout visits (bottom) at 5-9 months of age (blue) and 18-20 months of age (green) in *App$^{NL\text{-}G\text{-}F}$* (C) and age-matched control (D) mice, respectively. The red dashed lines indicate the memory time span. 5-9 months of age: blue; 18-20 months of age: green; shaded regions: standard error of the mean (SEM).

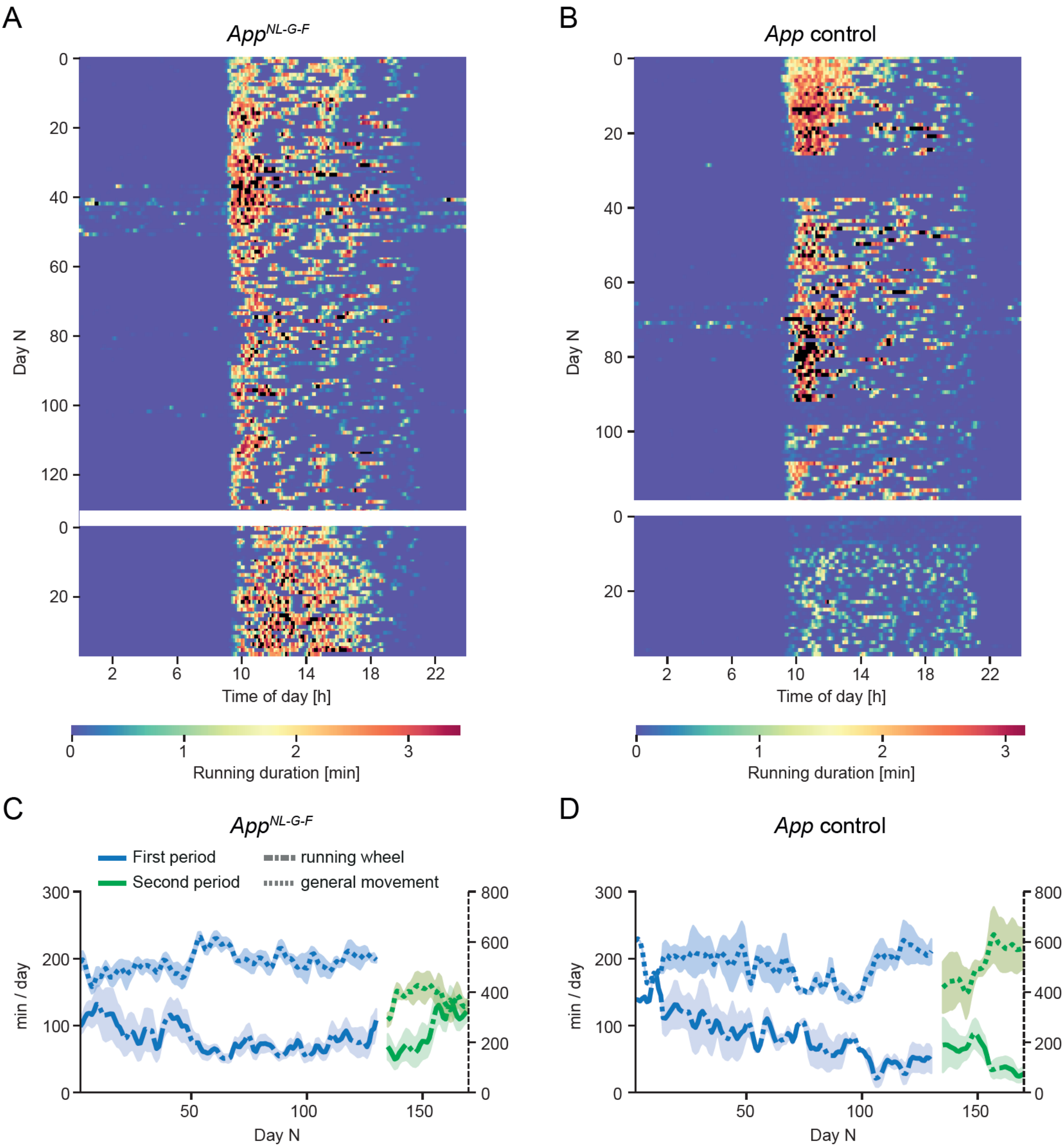

Figure S7. Locomotion patterns of ***App***$^{NL\text{-}G\text{-}F}$ **animals, related to Figure 6. (A,B)** Example ethograms showing the daily wheel-running activity of a single *App*$^{NL\text{-}G\text{-}F}$ (A) and age-matched control (B) mouse. The white gap demarcates the two testing periods of *App*$^{NL\text{-}G\text{-}F}$ animals – the top portion at 5-9 months old and the bottom portion at 18-20 months old. **(C,D)** Average general locomotion (dotted) and wheel-running (dash-dotted) behaviours in *App*$^{NL\text{-}G\text{-}F}$ (C) and age-matched control (D) mice. 5-9 months of age: blue; 18-20 months of age: green; shaded regions: standard error of the mean (SEM).

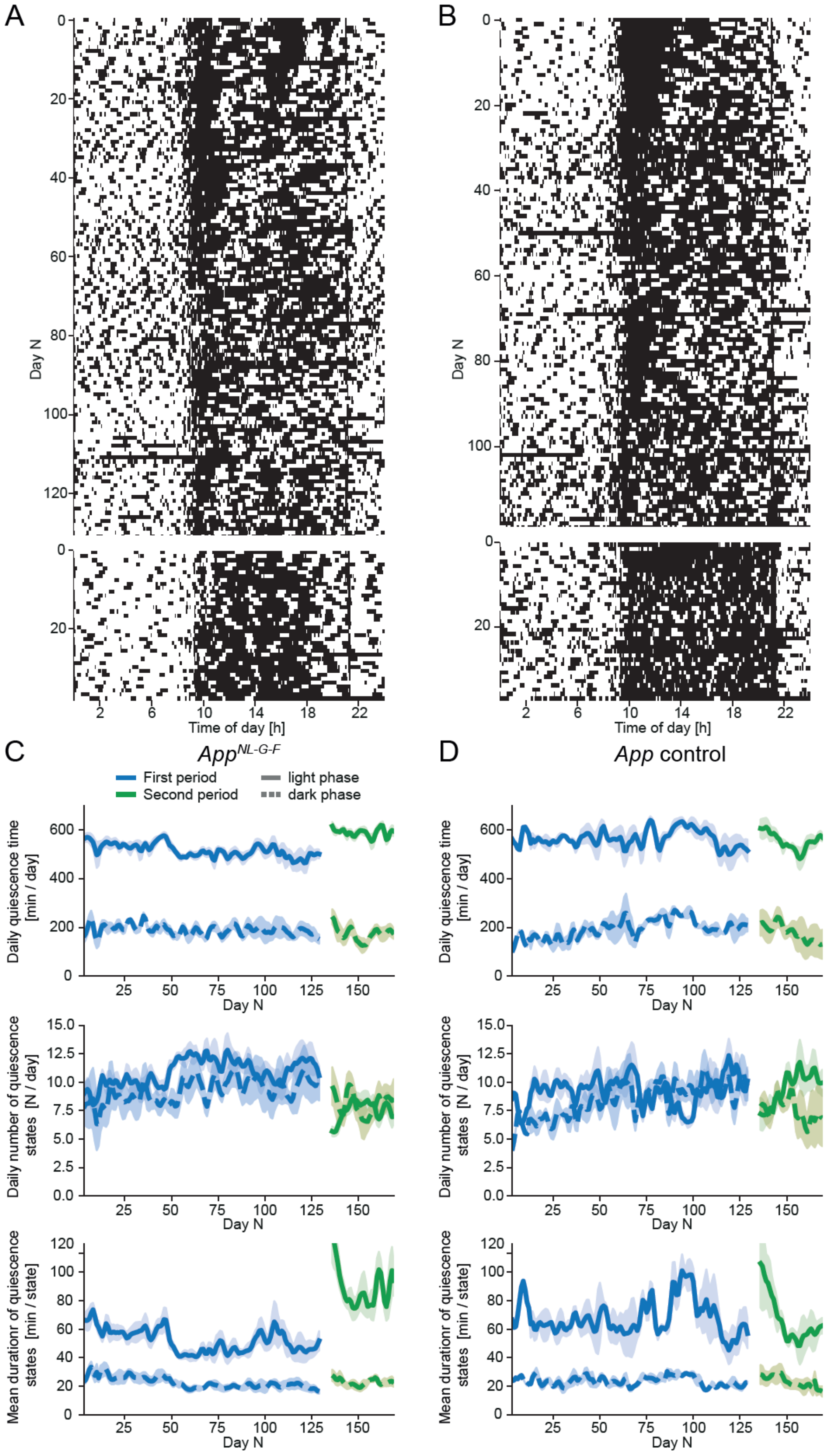

Figure S8. Quiescence patterns of *App*$^{NL-G-F}$ animals, related to Figure 6. (A,B) Example ethograms showing quiescence states of a single *App*$^{NL-G-F}$ (A) and an age-matched control (B) mouse. White and black regions indicate quiescence and mobile intervals, respectively. The white gap demarcates the two testing periods of *App*$^{NL-G-F}$ mice – the top portion at 5-9 months old and the bottom portion at 18-20 months old. **(C,D)** The total daily average time spent in quiescence (top), the daily average number of quiescence states (middle) and their average duration (bottom) in *App*$^{NL-G-F}$ (C) and age-matched control (D) mice. Solid and dashed lines correspond to light and dark phases, respectively. 5-9 months of age: blue; 18-20 months of age: green; shaded regions: standard error of the mean (SEM).

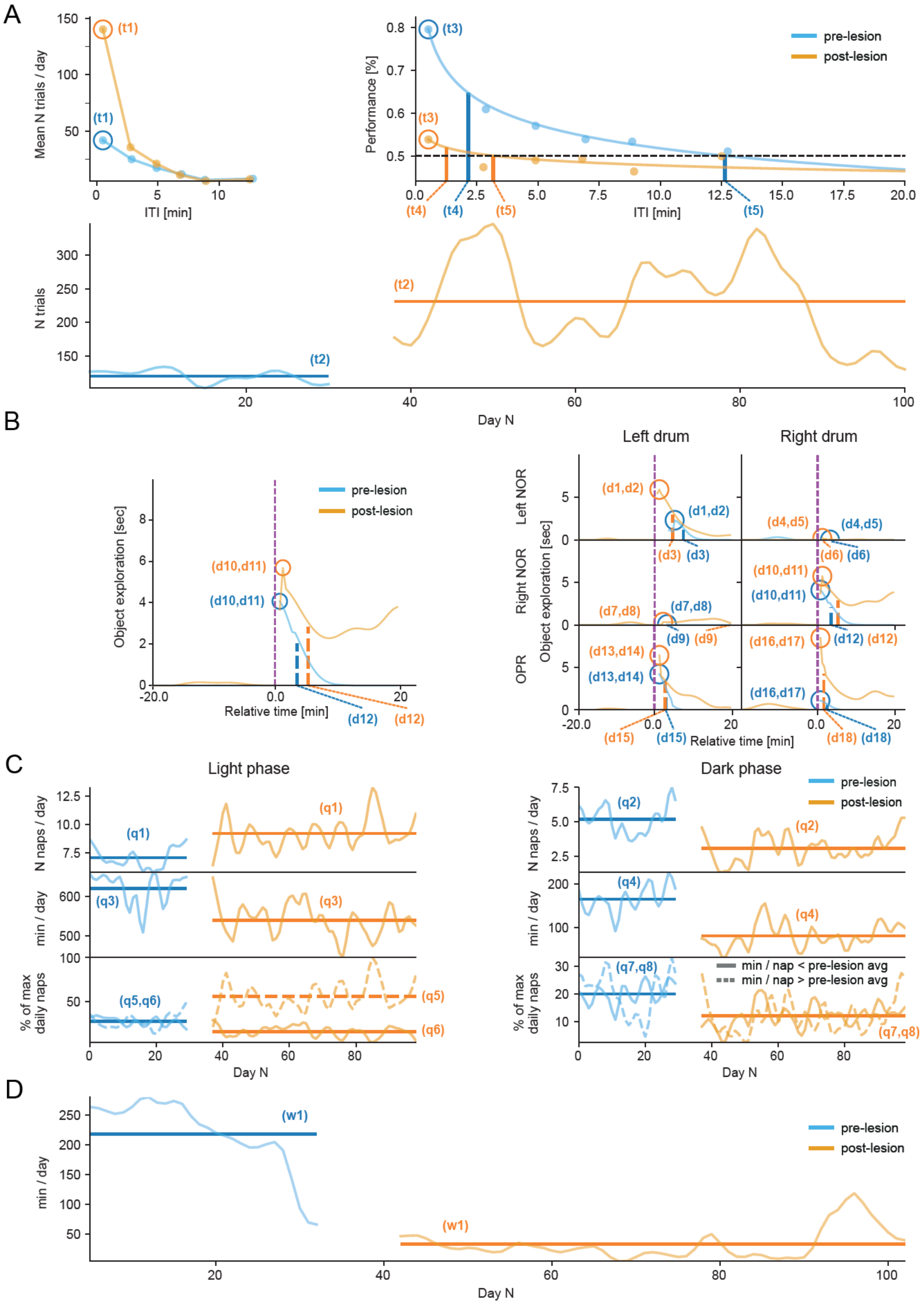

Figure S9. Behavioural features used in unsupervised clustering, related to Figure 7. Pre- (blue) and post-lesion (orange) features were considered independent during clustering. **(A)** T-maze features. (t1) Maximal daily-average trial frequency w.r.t. ITI. (t2) Average daily trial frequency. (t3) Maximal performance w.r.t. ITI [%]. (t4) ITI at a half-point between maximal and 50% performance [min]. (t5) ITI at 50% performance [min]. **(B)** NOR/OPR features. (d1, d4, d7, d10, d13, d16) Average maximal exploration after drum change [sec]. (d2, d5, d8, d11, d14, d17). The average elapsed time between drum change and maximal exploration [min]. (d3, d6, d9, d12, d15, d18) average elapsed time between drum change and half-maximal exploration [min]. The 3 NOR/OPR features were extracted from both drums for left NOR, right NOR and OPR drum changes, resulting in 18 total NOR/OPR features per animal. **(C)** Quiescence features. (q1,q2) Average daily quiescence frequency. (q3,q4) Average daily quiescence time [min]. (q5,q6) Percent of maximal naps with duration below pre-lesion average [%]. (q7,q8) Percent of maximal naps with duration above pre-lesion average [%]. The four quiescence features were extracted from light and dark phases, resulting in 8 total quiescence features per animal. **(D)** Running-wheel features. (w1) Average daily time running on the wheel [min].

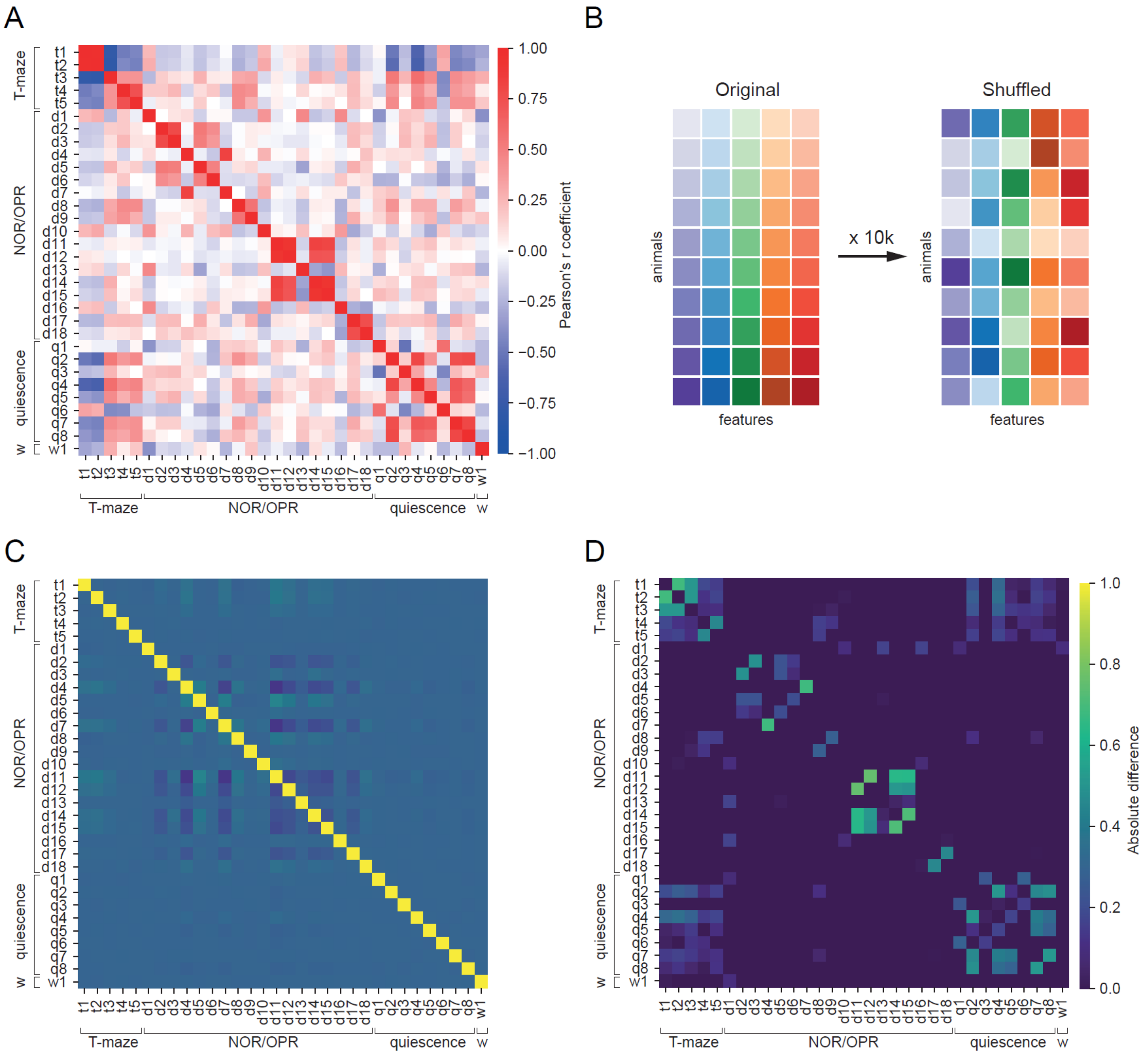

Figure S10. Correlations between all 32 behavioural features used in clustering, related to STAR Methods. (A) Pairwise Pearson's r correlation. Each (x,y) entry in the matrix represents the calculated Pearson's r coefficient between features x and y. **(B)** Schematic of random shuffling simulations. In each simulation, the features are shuffled among animals but within the same feature type (colour column). The pairwise Pearson's r correlation is calculated between each feature pair of the shuffled matrix as in (A). The resultant 10,000 shuffled correlation matrices are then used to calculate the 95th percentile of the absolute Pearson's correlation for each feature pair to produce a matrix in part (C). The absolute values of Pearson's r are taken here as we only care about the magnitude, not the sign, of all correlations. **(C)** Pearson's correlation of randomly shuffled features, calculated as the absolute 95th percentile from 10,000 random shuffles. **(D)** Difference between absolute plot (A) and plot (C). Since both plots consist of positive (absolute) values, positive scores in (D) feature above chance level correlations.

Table S1. Mouse groups tested in the smart-Kage, related to Figure 2 and 6.

Group name	Lesion / genotype	Number of mice	Age at the start of smart-Kage experiment (weeks)	Gender
Batch #1	Sham	4	10	M
	mEC	2	10	M
Batch #2	Sham	5	16	M
	mEC	2	16	M
	HP	5	16	M
Batch #3	unlesioned	10	16	M
App^{NL-G-F}	WT control	3	22 (1st period)<br>72 (2nd period)	M
	mutant KI	5	22-24&39<br>(1stperiod) 72-80<br>(2nd period)	M

Table S2. Behavioural features used in unsupervised clustering, related to Figure 7.

Category	Features			
T-maze (Figure S10a)	t1	maximal daily-average trial frequency (number of times the mouse attempts to drink in the T-maze) w.r.t. ITI		
	t2	daily average trial frequency (number of times the mouse attempts to drink in the T-maze)		
	t3	maximal performance w.r.t. ITI [%]		
	t4	ITI at a half-point between maximal and 50% performance [min]		
	t5	ITI at 50% performance [min]		
NOR / OPR (Figure S10b)	d1	LEFT NOR	LEFT DRUM	daily avg. maximal exploration after drum change [sec]
	d2			daily avg. elapsed time between drum change and max exploration [min]
	d3			daily avg. elapsed time between drum change and half-max expl. [min]
	d4		RIGHT DRUM	daily avg. maximal exploration after drum change [sec]
	d5			daily avg. elapsed time between drum change and max exploration [min]
	d6			daily avg. elapsed time between drum change and half-max expl. [min]
	d7	RIGHT NOR	LEFT DRUM	daily avg. maximal exploration after drum change [sec]
	d8			daily avg. elapsed time between drum change and max exploration [min]
	d9			daily avg. elapsed time between drum change and half-max expl. [min]
	d10		RIGHT DRUM	daily avg. maximal exploration after drum change [sec]
	d11			daily avg. elapsed time between drum change and max exploration [min]
	d12			daily avg. elapsed time between drum change and half-max expl. [min]
	d13	OPR	LEFT DRUM	daily avg. maximal exploration after drum change [sec]
	d14			daily avg. elapsed time between drum change and max exploration [min]
	d15			daily avg. elapsed time between drum change and half-max expl. [min]
	d16		RIGHT DRUM	daily avg. maximal exploration after drum change [sec]
	d17			daily avg. elapsed time between drum change and max exploration [min]
	d18			daily avg. elapsed time between drum change and half-max expl. [min]
Quiescence (Figure S10c)	q1	LIGHT PHASE	daily average quiescence frequency (number of quiescent intervals)	
	q2	DARK PHASE		
	q3	LIGHT PHASE	daily average quiescence time [min]	
	q4	DARK PHASE		
	q5	LIGHT PHASE	avg. percent of maximal naps with duration **BELOW** pre-lesion average [%]	
	q6		avg. percent of maximal naps with duration **ABOVE** pre-lesion average [%]	
	q7	DARK PHASE	avg. percent of maximal naps with duration **BELOW** pre-lesion average [%]	
	q8		avg. percent of maximal naps with duration **ABOVE** pre-lesion average [%]	
Running wheel (Figure S10d)	w1	daily average time of running on the wheel [min]		
